# Chromatin-remodelling factor Brg1 regulates myocardial proliferation and regeneration in zebrafish

**DOI:** 10.1038/ncomms13787

**Published:** 2016-12-08

**Authors:** Chenglu Xiao, Lu Gao, Yu Hou, Congfei Xu, Nannan Chang, Fang Wang, Keping Hu, Aibin He, Ying Luo, Jun Wang, Jinrong Peng, Fuchou Tang, Xiaojun Zhu, Jing-Wei Xiong

**Affiliations:** 1 Institute of Molecular Medicine, Peking University, Beijing 100871, China; 2 Beijing Key Laboratory of Cardiometabolic Molecular Medicine, Peking University, Beijing 100871, China; 3 State Key Laboratory of Natural and Biomimetic Drugs, Beijing 100871, China; 4 Biodynamic Optical Imaging Center, Peking University, Beijing 100871, China; 5 College of Life Sciences, Peking University, Beijing 100871, China; 6 School of Life Sciences, University of Science and Technology of China, Hefei 230026, China; 7 Hefei National Laboratory for Physical Sciences at the Microscale, University of Science and Technology of China, Hefei 230026, China; 8 Department of Biomedical Engineering, College of Engineering, Peking University, Beijing 100871, China; 9 Institute of Medicinal Plant Development, Chinese Academy of Medical Sciences, Beijing 100193, China; 10 Peking Union Medical College, Beijing 100730, China; 11 College of Animal Sciences, Zhejiang University, Hangzhou 310058, China

## Abstract

The zebrafish possesses a remarkable capacity of adult heart regeneration, but the underlying mechanisms are not well understood. Here we report that chromatin remodelling factor Brg1 is essential for adult heart regeneration. Brg1 mRNA and protein are induced during heart regeneration. Transgenic over-expression of dominant-negative *Xenopus Brg1* inhibits the formation of BrdU^+^/Mef2C^+^ and Tg(*gata4*:EGFP) cardiomyocytes, leading to severe cardiac fibrosis and compromised myocardial regeneration. RNA-seq and RNAscope analyses reveal that inhibition of Brg1 increases the expression of cyclin-dependent kinase inhibitors such as *cdkn1a* and *cdkn1c* in the myocardium after ventricular resection; and accordingly, myocardial-specific expression of *dn-xBrg1* blunts myocardial proliferation and regeneration. Mechanistically, injury-induced Brg1, via its interaction with Dnmt3ab, suppresses the expression of *cdkn1c* by increasing the methylation level of CpG sites at the *cdkn1c* promoter. Taken together, our results suggest that Brg1 promotes heart regeneration by repressing cyclin-dependent kinase inhibitors partly through Dnmt3ab-dependent DNA methylation.

## Abstract (web summary)

The adult zebrafish heart is capable of regeneration but the molecular mechanisms are poorly understood. Here the authors show that chromatin remodeling factor Brg1 represses cyclin-dependent kinase inhibitors to promote myocardial regeneration.

The high mortality and morbidity following myocardial infarction is a public health problem worldwide. Myocardial infarction results in the loss of billions of cardiomyocytes in heart failure patients while myocardial regeneration is severely limited. Various cell-based and cell-free strategies are being explored for promoting heart regeneration in animal models and human patients^1^,^2^,^3^. However, the efficacy of cardiac cell-based therapy is still uncertain, with frequent occurrence of engraftment-induced arrhythmia, so the clinical implications remain unclear^4^. In contrast, lower vertebrates such as zebrafish can perfectly regenerate the injured heart by cardiomyocyte dedifferentiation and proliferation^5^,^6^,^7^,^8^. Although cardiac regeneration after ventricular resection occurs in mouse neonatal heart at 1 day after birth, this regenerative capacity is lost within 7 days after birth^9^, suggesting that regenerative potential is gradually lost during mouse heart development and maturation. In spite of the very limited regenerative capacity, mammalian cardiomyocytes are able to divide and renew in adulthood^10^,^11^,^12^. Therefore, harnessing the mechanisms underlying zebrafish heart regeneration may provide insights into mammalian heart regeneration and have therapeutic applications.

ATP-dependent chromatin remodelling is involved in controlling chromatin structure that in turn regulates many physiological and pathological processes. Instead of covalently modifying DNA or histones, the SWI/SNF (SWI/sucrose non-fermentable)-like complex, a member of the family of ATP-dependent chromatin-remodelling complexes, uses energy from ATP hydrolysis, and regulates gene transcription by rearranging nucleosome positions and histone–DNA interactions, and thus facilitates the transcriptional activation or repression of targeted genes^13^. The SWI/SNF complex contains >10 components, of which brahma-related gene 1 (BRG1, or SMARCA4) is one of the central ATPase catalytic subunits. This complex plays an important role in the development of the central nervous system, thymocytes, heart and other organs. *Brg1* is essential for zygote genome activation^14^, erythropoiesis^15^, cardiac development^16^,^17^ and neuronal development^18^,^19^. Other members of the mammalian SWI/SNF complex are also required for heart morphogenesis, including *Baf60c* (ref. 20), *Baf180* (ref. 21) and *Baf250a* (ref. 22). In particular, *Brg1* controls cardiovascular development in a time- and tissue-specific manner. *Brg1* deletion in mice results in embryonic lethality before implantation^23^. Endothelial and endocardial depletion of *Brg1* results in embryonic death and failure of myocardial trabeculation around E10.5 in mice^16^. Mice with myocardial depletion of *Brg1* die around E11.5 due to thin compact myocardium and the absence of the interventricular septum^17^. In embryos, *Brg1* promotes cardiomyocyte proliferation by maintaining *Bmp10* and suppressing *p57*^*kip2*^ (*cdkn1c*) expression^17^. Brg1 suppresses *Ask1* and *Cdkn1a* to inhibit apoptosis and promote proliferation of neural crest cells^24^. Besides its effects on cardiomyocyte proliferation, *Brg1* also controls α and β myosin heavy-chain switching in the embryonic and adult hearts under hypertrophic stimulations^17^. The function of *Brg1* in heart development is evolutionarily conserved between zebrafish and mammals. Mutation of *brg1* in zebrafish causes cardiac hypoplasia and severe arrhythmia with abnormal expression patterns of several heart-specific genes^25^. Besides its functions in organ development, *Brg1* is also required for hair regeneration and epidermal repair. *Brg1* knockdown impairs bulge cell proliferation partly through elevating the cyclin-dependent kinase inhibitor p27^Kip1^ (*cdkn1b*)^26^.

Although several subunits of the SWI/SNF complex are essential for cardiac development, little is known about how this complex orchestrates zebrafish heart regeneration at the chromatin level. To address this important question, we set out to determine whether and how the disruption of Brg1 affects zebrafish heart regeneration. Here we find that *brg1* mRNA and protein are induced during the course of cardiac regeneration, and inhibition of Brg1 leads to severe cardiac fibrosis and compromised myocardial regeneration. Myocardial-specific expression of *dn-xBrg1* blunts myocardial proliferation and regeneration by increasing cell-cycle-dependent inhibitors in the myocardium. Furthermore, injury-induced Brg1 interacts with Dnmt3ab to suppress the expression of *cdkn1c* by increasing the methylation level of CpG sites at the *cdkn1c* promoter. This study has gained molecular insights of Brg1 into zebrafish heart regeneration and has shed light on potential intervention of this complex for promoting heart repair and regeneration in humans.

## Results

### *Brg1* is upregulated after ventricular apex amputation

**Figure 1:**
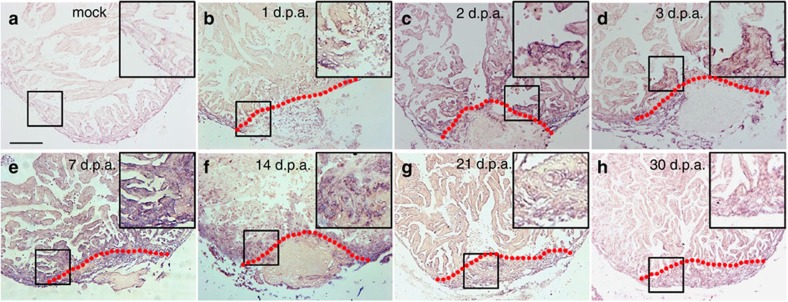
*brg1* is upregulated during cardiac regeneration in zebrafish. *In situ* hybridization was performed on paraffin sections of mock-operated zebrafish (**a**) and those with amputated ventricular apexes (**b**–**h**) at the indicated time points using a digoxigenin-labelled anti-sense *brg1* RNA probe. Note induced expression of *brg1* in the injured heart from 1 to 14 d.p.a. (**b**–**f**). Dashed lines mark the resection sites; the right upper corner is high-magnification image of the framed area; similar results were confirmed by performing three independent experiments. Scale bar, 100 μm.

In spite of great efforts in many laboratories, it remains challenging to induce mammalian cardiomyocytes to re-enter mitosis by either activating a single cyclin-dependent kinase or inactivating a single cyclin-dependent kinase inhibitor^27^,^28^,^29^. We hypothesized that a global epigenetic change might occur during zebrafish heart regeneration and so manipulating epigenetic programmes might be an efficient means of inducing mammalian cardiomyocytes to re-enter mitosis. To evaluate the functions of the SWI/SNF complex during zebrafish cardiac regeneration, we performed *in situ* hybridization screens to identify expression patterns of the complex components after ventricular apex amputation. Interestingly, several members of this complex (*brg1*, *baf60c and baf180*) were induced during regeneration (Fig. 1 and Supplementary Fig. 1). *brg1* transcripts were upregulated as early as 2 days post amputation (d.p.a.), peaked and concentrated proximal to the injury site at 3, 7 and 14 d.p.a., and became undetectable at 30 d.p.a., when regeneration was nearly complete (Fig. 1). These data support our hypothesis that the chromatin-remodelling BAF complex is associated with zebrafish heart regeneration.

**Figure 2:**
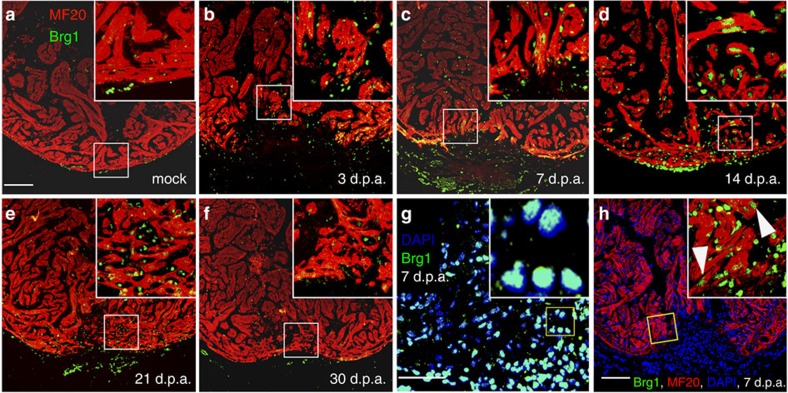
Brg1 is activated in multiple types of cells during cardiac regeneration in zebrafish. (**a**–**f**) Immunofluorescence staining of Brg1 and cardiac sarcomere myosin heavy chain (MF20) was performed on paraffin sections of mock-operated zebrafish (**a**) and those with amputated ventricular apexes (**b**–**f**) at the indicated time points. The right upper corners are high-magnification images of the frame area in a–f, showing Brg1 co-localization in MF20-positive myocytes. (**g**) Co-staining of Brg1 and 4,6-diamidino-2-phenylindole (DAPI) in paraffin sections of amputated apexes at 7 d.p.a. The right upper corner is high-magnification image of the framed area. (**h**) Immunofluorescence staining of Brg1 and MF20 of amputated heart at 7 d.p.a., showing the co-localization of Brg1 and MF20. The right upper corner is high-magnification image of the framed area. These data were confirmed by performing three independent experiments. Scale bars, 100 μm.

Next, we investigated where Brg1 proteins were expressed in the cardiac cells of injured hearts using immunofluorescence staining. Consistent with its mRNA expression pattern, Brg1 was almost undetectable in the mock-operated hearts but highly induced around the injured area at 3, 7 and 14 d.p.a.; it then declined from 21 to 30 d.p.a. (Fig. 2a–f). Co-staining with 4,6-diamidino-2-phenylindole showed that Brg1 was located in the nuclei (Fig. 2g), in accord with the fact that Brg1 is a nuclear ATPase of the SWI/SNF complex^30^. In addition, the specificity of this anti-human BRG1 antibody was confirmed by its recognition of zebrafish/frog Brg1 proteins in *brg1* morphants and *dn-xBrg1*-over-expressing embryos, as well as pull down of endogenous zebrafish Brg1 protein by immunoprecipitation (Supplementary Figs 2a and 3b). To identify Brg1-expressing cells, we carried out co-immunostaining for Brg1 and myocardium-specific myosin heavy chain (MF20), endocardial/endothelial reporter Tg(*flk1*:nucEGFP), macrophage/neutrophil reporter Tg(*coronin1a*:EGFP), epicardial reporter Tg(*tcf21*:DsRed) or myocardial reporter Tg(*gata4*:EGFP). Brg1 was detected within MF20-positive myocardial cells at 7 d.p.a. (Fig. 2h). Furthermore, Brg1 was co-localized in the injury site with Tg(*flk1*:nucEGFP)-positive endocardium (Supplementary Fig. 2b), Tg(*coronin1a*:EGFP)-positive macrophages/neutrophils (Supplementary Fig. 2c), Tg(*tcf21*:DsRed)-positive epicardium (Supplementary Fig. 2d) and Tg(*gata4*:EGFP)-positive myocardium (Supplementary Fig. 2e). Additional analyses showed that about 20–30% of Brg1^+^ cells were co-localized with MF20-positive myocardium (Supplementary Fig. 2g,h) or with flk1:nucEGFP-positive endocardium (Supplementary Fig. 2i,j) from 3 to 14 d.p.a.; about 20–30% of Brg1^+^ cells were co-localized with tcf21:DsRed-positive epicardium from 7 to 21 d.p.a. (Supplementary Fig. 2k,l); and about 7% of Brg1^+^ cells were co-localized with coronin1a:EGFP-positive leukocytes (Supplementary Fig. 2m,n) from 7 to 14 d.p.a. Together, our data suggested that Brg1 is induced in multiple types of cells in the heart after ventricular apex amputation.

### Inhibition of Brg1 blocks heart regeneration in zebrafish

**Figure 3:**
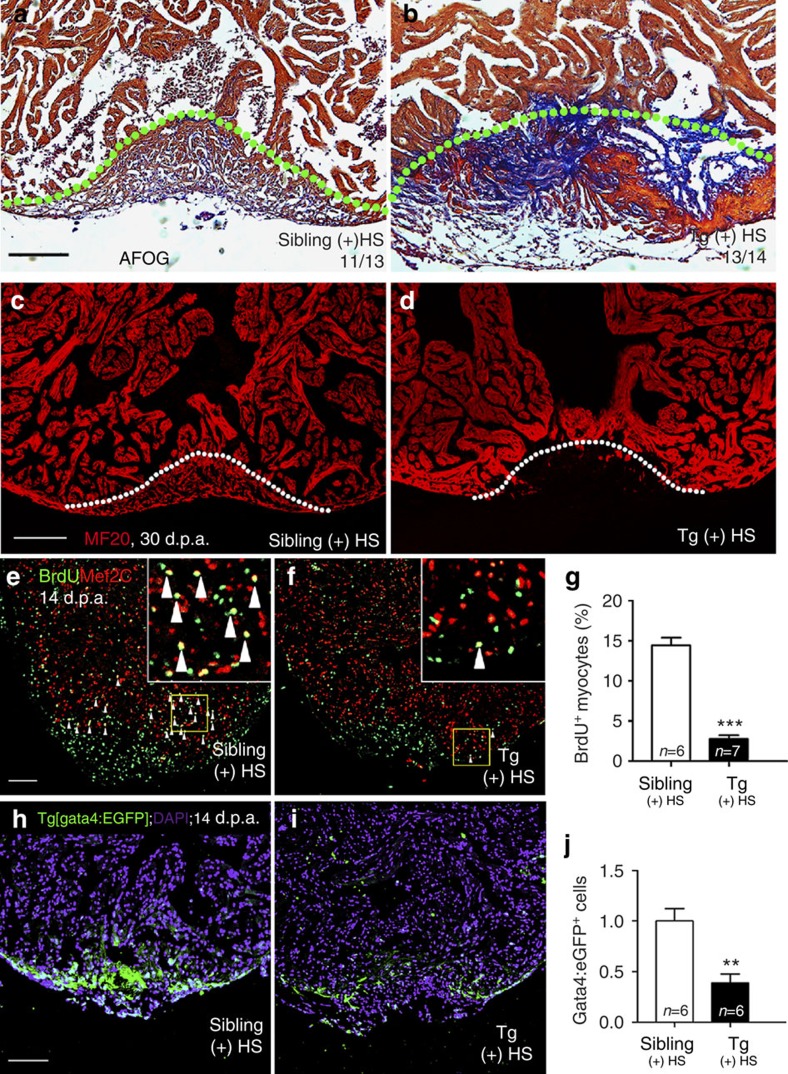
Inhibition of *brg1* impairs cardiac regeneration. (**a**–**d**) Representative sections from wild-type siblings (**a**,**c**) and Tg(*hsp70*:dn-xbrg1) (**b**,**d**) hearts at 30 d.p.a., evaluated by AFOG staining (**a**,**b**), and immunofluorescence staining with anti-myosin heavy chain (MF20) (**c**,**d**). Note massive fibrosis (**b**) and compromised myocardial regeneration (**d**) in Tg(*hsp70*:dn-xBrg1) hearts (tg). Dashed lines mark the resection site. (**e**–**g**) Paraffin sections of 14 d.p.a. regenerating heart of wild-type sibling (**e**) and Tg(*hsp70*:dn-xBrg1) (**f**) hearts co-stained for BrdU (green), Mef2C (red) and 4,6-diamidino-2-phenylindole (DAPI; blue). Higher-magnification images of areas in squares are shown in the upper-right corners, and Mef2C^+^/BrdU^+^ double-positive cardiomyocytes are indicated by arrowheads. (**g**) Percentages of Mef2C^+^/BrdU^+^ cardiomyocytes in the injured area (***P<0.001; *n*=6 for siblings and 7 for transgenic hearts; data are mean percentages±s.e.m., paired Student’s *t*-test). (**h**–**j**) Paraffin sections of 14 d.p.a. wild-type Tg(*gata4*:EGFP) sibling (**h**) and Tg(*hsp70*:dn-xbrg1; *gata4*:EGFP) (**i**) hearts stained with anti-EGFP and DAPI. The average of fluorescence intensity was calculated using Imaris software (**j**) (**P<0.01; *n*=6; data are mean percentages±s.e.m.; paired Student’s *t*-test). Scale bars, 100 μm.

Elevated expression of *brg1* mRNA and protein following ventricular apex amputation suggested that *brg1* might participate in regeneration. To determine the function of Brg1, we applied a dominant-negative *Xenopus Brg1* (*dn-xBrg1*) that carries a K770T771-to-A770A771 mutation in the ATP-binding pocket^19^. This mutant Brg1 protein can bind to the other components of the SWI/SNF complex but its ATPase domain is disrupted and thus plays a dominant-negative role. Brg1 is highly conserved with 84% identity in amino acids between zebrafish and frog, and the ATP-binding pocket, which is mutated in the Xenopus dominant-negative Brg1, is identical between the two proteins. We generated a Tg(*hsp70*:dn-xbrg1) transgenic strain in which the *dn-xBrg1* was driven by the zebrafish heat shock promoter^31^. As expected, over-expression of Xenopus *dn-xBrg1* inhibited Brg1 function and caused mutant heart to display stenosis shown by myocardial markers (*cmlc2*, *vmhc*, *amhc* and *nppa*) and had slight expanded expression domains of *bmp4* and *tbx2b* while decreased expression of *notch1b* in the atrioventricular canal as those in chemical-induced zebrafish *brg1* mutant embryos at 48 or 60 h.p.f. (ref. 25; Supplementary Fig. 3). We then performed ventricular amputation in Tg(*hsp70*:dn-xBrg1) zebrafish and their wild-type siblings followed by heating at 37 °C for 30 min daily from 5 to 30 d.p.a. Acid fuchsin orange G (AFOG) stain for extracellular matrix showed increased fibrosis and lack of sealing of the wound in *dn-xBrg1* transgenic hearts (Fig. 3b and Supplementary Fig. 4c) compared with wild-type siblings (Fig. 3a and Supplementary Fig. 4a) at 30 d.p.a. Myocardial regeneration, visualized by MF20 immunostaining, was defective in *dn-xBrg1* transgenic hearts (Fig. 3d and Supplementary Fig. 4d,e) compared with the fully regenerated myocardium in wild-type siblings (Fig. 3c and Supplementary Fig. 4b,e) at 30 d.p.a. After heat shock from 5 to 30 d.p.a., we stopped heat shock treatment and examined the hearts at 60 d.p.a. by AFOG and MF20 staining. Heat shock caused comparable lethality between *dn-xBrg1* transgenic zebrafish and their wild-type siblings (Supplementary Fig. 4f). We still found cardiac fibrosis and compromised myocardial regeneration in *dn-xBrg1* transgenic hearts (Supplementary Fig. 5c,d) compared with wild-type sibling hearts (Supplementary Fig. 5a,b), suggesting that inhibiting Brg1 caused permanent defects in heart regeneration. On the other hand, conditional over-expression of wild-type *brg1* had no effects on myocardial proliferation (Supplementary Fig. 6), suggesting that other members of SWI/SNF complex are also required for this process. Taken together, these data demonstrated that inhibition of Brg1 caused a regenerative defect in the zebrafish heart and so Brg1 is required for cardiac regeneration.

Previous studies suggest that regenerated cardiomyocytes originate from the de-differentiation and proliferation of cardiomyocytes near injury site^7^,^32^. Thus, we evaluated whether inhibition of Brg1 had any effect on the de-differentiation of cardiomyocytes after injury. We found comparable de-differentiation of cardiomyocytes in the injury site, as measured disassembly of cardiac sarcomeres by transmission electron microscopy (Supplementary Fig. 7a–d), or disrupted Z-disks labelled by cypher-EGFP fusion protein^33^ (Supplementary Fig. 7e–l) in both wild-type sibling and Tg(*hsp70*:dn-xbrg1) transgenic hearts at 14 d.p.a. We next compared the index of cardiomyocyte proliferation in Tg(*hsp70*:dn-xBrg1) and wild-type sibling hearts at 14 d.p.a. after heat shock by quantifying the percentage of BrdU and myocardial marker Mef2C double-positive nuclei near the injury area. Compared with ∼14% BrdU^+^/Mef2C^+^ cardiomyocytes in wild-type sibling hearts at 14 d.p.a. (Fig. 3e,g), we found only 2.4% proliferating cardiomyocytes in Tg(*hsp70*:dn-xBrg1) hearts (Fig. 3f,g), which was further confirmed by measuring PCNA^+^/Mef2C^+^ proliferating cardiomyocytes (Supplementary Fig. 8d–f). In addition, both Tg(*hsp70*:dn-xBrg1) transgenic and wild-type sibling hearts, without heat shock treatments, had similar BrdU^+^/Mef2C^+^ proliferating cardiomyocytes (Supplementary Fig. 8a–c), suggesting the minimal effects of heat shock on the index of proliferating cardiomyocytes. As previously reported^7^,^34^, Gata4-positive cardiomyocytes appeared near the injured area in wild-type Tg(*gata4*:EGFP) hearts at 14 d.p.a. (Fig. 3h). However, there were markedly fewer Gata4:EGFP-positive cardiomyocytes in Tg(*hsp70*:dn-xBrg1; *gata4*:EGFP) hearts at 14 d.p.a. (Fig. 3i,j). These data suggested that Brg1 is essential for cardiomyocyte proliferation and regeneration after ventricular apex amputation in zebrafish.

The endocardium and epicardium are also known to be activated after ventricular amputation^35^. Immunostaining revealed that epicardial and endocardial marker Raldh2 (Supplementary Fig. 9a–c) and epicardial reporter Tg(*tcf21*:DsRed) (Supplementary Fig. 9d–f) were similarly expressed in wild-type sibling and Tg(*hsp70*:dn-xBrg1) hearts, suggesting that the organ-wide activation of endocardium and epicardium was not affected after inhibition of Brg1. In addition, coronary vessel regeneration accompanied myocardial regeneration after ventricular resection^36^. By labelling coronary vessels and endocardium with the Tg(*flk1*:EGFP) transgene, we found fewer flk1^+^ endothelium and endocardium in the injured area of Tg(*hsp70*:dn-xBrg1) hearts than in wild-type sibling hearts (Supplementary Fig. 9g–i), suggesting that Brg1 is also critical for the formation of newly regenerated coronary vessels since the Raldh2^+^ endocardium is less affected during heart regeneration.

### Brg1 represses cyclin-dependent kinase inhibitor genes

**Figure 4:**
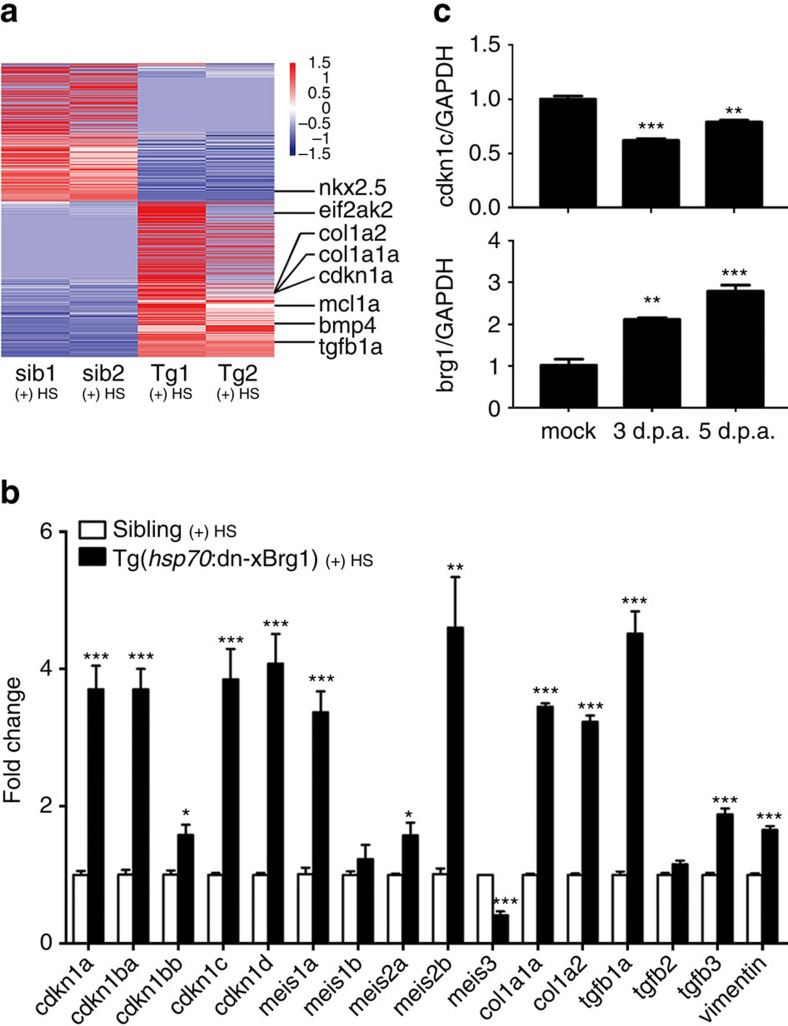
Transgenic inhibition of Brg1 induces expression of cyclin-dependent kinase inhibitors. (**a**) Heat map of *Z*-score values showing genes differentially expressed between Tg(*hsp70*:dn-xBrg1) (tg1 and tg2) and wild-type sibling (sib1 and sib2) hearts. The FPKM (fragments per kilobase of exon per million fragments mapped) value of each gene was normalized using *Z*-scores. Genes were ranked by the mean *Z*-scores in the highest-expression group. (**b**) Tg(*hsp70*:dn-xBrg1) and wild-type sibling zebrafish were heat-shocked daily from 5 to 14 d.p.a., and total RNA was isolated from their hearts at 14 d.p.a. Quantitative PCR showed that the cdkn and meis genes, as well as fibrotic markers (*col1a1a*, *col1a2*, *tgfb1a*, *tgfb2*, *tgfb3* and *vimentin*) were upregulated in transgenic hearts (*P<0.05, ***P<0.001; data are mean fold changes after normalized to GAPDH and expressed as mean±s.e.m.; paired Student’s *t*-test). (**c**) Quantitative PCR showed higher expression of *brg1* but lower expression of *cdkn1c* in wild-type hearts at 3 and 5 d.p.a. than mock hearts, suggesting a repressive role of *brg1* in regulating *cdkn1c*. GAPDH was used to normalize the RNA level (**P<0.01, ***P<0.001; data are mean±s.e.m.; one-way analysis of variance followed by Dunnett’s multiple comparison test, mock served as control).

To decipher the molecular mechanisms underlying the regulation of myocardial proliferation by Brg1, we performed RNA-seq to compare the transcriptomes in Tg(*hsp70*:dn-xBrg1) and wild-type sibling hearts after heat shock daily from 5 to 14 d.p.a. We obtained ∼4 million 100 bp pair-end reads for each sample. Further bioinformatics analyses revealed that 1,204 genes were upregulated while 1,092 genes were downregulated after the inhibition of Brg1 (Supplementary Data Sets 1 and 2). The genes with a twofold difference were selected for further analysis. *cdkn1a* was one of the upregulated genes in the Tg(*hsp70*:dn-xBrg1) hearts (Fig. 4a). Since Brg1 inhibition had a pronounced effect on myocardial proliferation and fibrosis in Tg(*hsp70*:dn-xBrg1) hearts, we focused on the genes regulating cell-cycle progression and proliferation as well as cardiac fibrosis. Using reverse transcription–PCR (RT–PCR), we found that other CDK inhibitors *cdkn1a*, *cdkn1ba*, *cdkn1bb*, *cdkn1c and cdkn1d* were also increased in the *dn-xBrg1* transgenic heart at 14 d.p.a. (Fig. 4b). A previous study reported that Meis1 is required for the transcriptional activation of CDK inhibitors (*cdkn2a*, *cdkn2b* and *cdkn1a*) in mice^37^. Consistently, *meis1a*, *meis2a* and *meis2b* were induced in the *dn-xBrg1* transgenic heart (Fig. 4b). In addition, several fibrotic genes *col1a1a*, *col1a2*, *tgfb1a*, *tgfb3* and *vimentin* were also upregulated in the *dn-xBrg1* transgenic heart (Fig. 4b). Importantly, *brg1* was induced while *cdkn1c* was reciprocally repressed in the early phases of regeneration (Fig. 4c). Taken together, our data suggested that Brg1 normally represses the expression of CDK inhibitors for priming heart regeneration in zebrafish.

**Figure 5:**
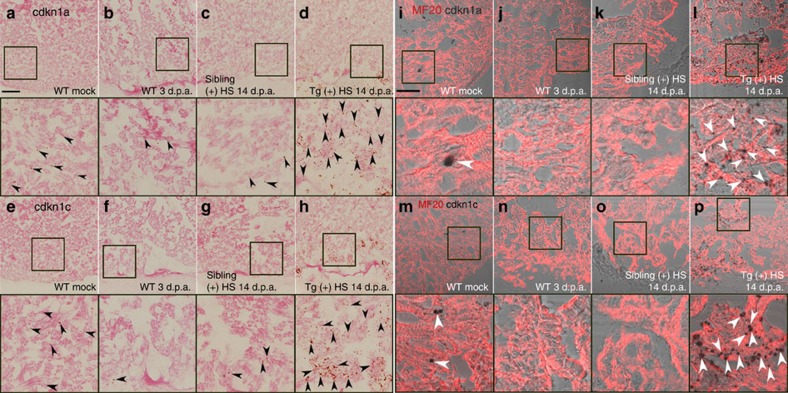
*cdkn1a* and *cdkn1c* are induced and enriched in the myocardium of Tg(*hsp70*:dn-xBrg1) transgenic hearts. (**a**–**h**) RNAscope *in situ* hybridization analysis with *cdkn1a* (**a**–**d**) and *cdkn1c* (**e**–**h**) probes on frozen sections of uninjured wild-type (WT) hearts (**a**,**e**), injured WT hearts at 3 d.p.a. (**b**,**f**), injured WT sibling hearts at 14 d.p.a. (**c**,**g**) and injured Tg(*hsp70*:dn-xBrg1) transgenic hearts at 14 d.p.a. (**d**,**h**). Note the robust induction of *cdkn1a* (**d**) and *cdkn1c* (**h**) induction in Tg(*hsp70*:dn-xBrg1) transgenic hearts compared with WT sibling hearts at 14 d.p.a. after heat shock. Black arrowheads indicate the *cdkn1a* or *cdkn1c* signals. The panels below **a**–**h** are higher-magnification images of areas in squares of **a**–**h**. (**i**–**p**) Bright-field images of *cdkn1a* (**i**–**l**) and *cdkn1c* (**m**–**p**) expression by RNAscope merged with immunostaining signal images of MF20 on frozen sections of uninjured WT hearts (**i**,**m**), injured WT hearts at 3 d.p.a. (**j**,**n**), injured WT sibling hearts at 14 d.p.a. (**k**,**o**) and injured Tg(*hsp70*:dn-xbrg1) transgenic hearts at 14 d.p.a. (**i**,**p**). Higher-magnification images of squared areas of **i**–**p** are shown below their respective panels. Note that both *cdkn1a* (**i**) and *cdkn1c* (**m**) are normally expressed in cardiomyocytes in uninjured WT hearts, and that they are highly induced in MF20-positive cardiomyocytes of Tg(*hsp70*:dn-xBrg1) transgenic hearts (**l**,**p**) compared with WT sibling hearts (**k**,**o**) at 14 d.p.a. White arrowheads show *cdkn1a* or *cdkn1c* signals in cardiomyocytes. Tg, Tg(*hsp70*:dn-xBrg1); (+) HS, heat shock; Scale bars, 100 μm.

**Figure 6:**
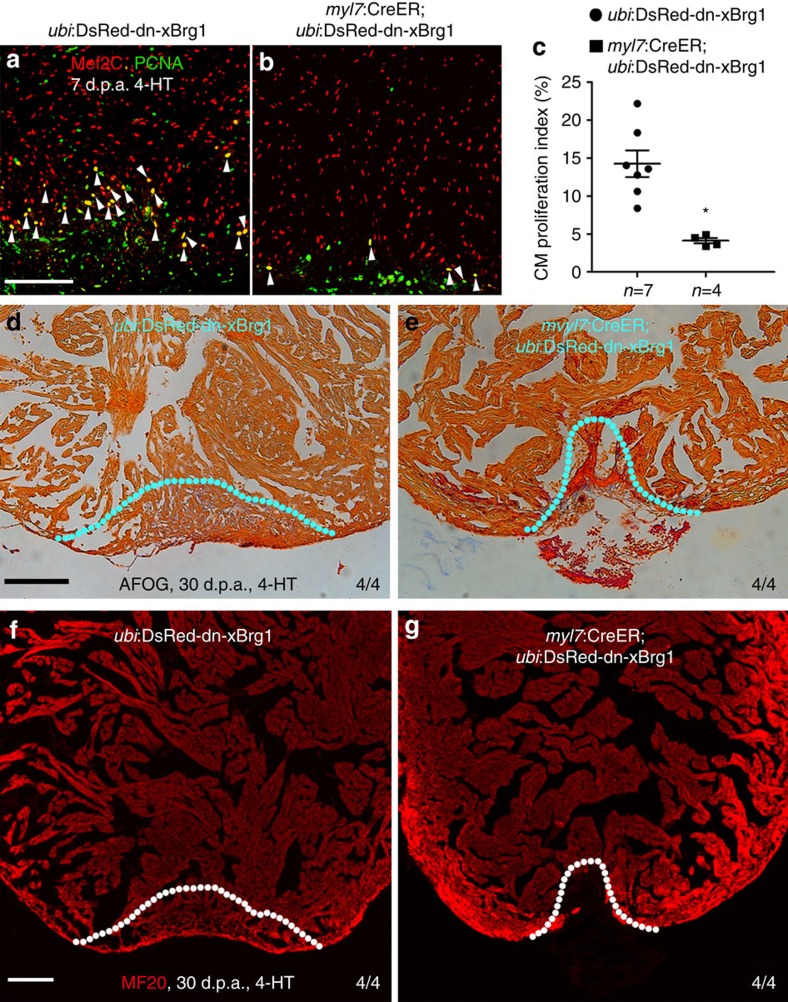
Myocardial-specific inhibition of Brg1 interferes heart regeneration. (**a**–**c**) PCNA^+^/Mef2C^+^ proliferating cardiomyocytes decreased in Tg(*myl7*:CreER; *ubi*:DsRed-dn-xBrg1) transgenic hearts (**b**) compared with control Tg(*ubi*:DsRed-dn-xBrg1) transgenic hearts (**a**) at 7 d.p.a. Statistics of cardiomyocyte proliferation index is shown (*P<0.05; data presented are mean±s.e.m.; paired Student’s *t*-test) (**c**). White arrowheads point to PCNA^+^/Mef2C^+^ proliferating cardiomyocytes; *n*, the number of hearts analysed; *ubi*:DsRed-dn-xBrg1 stands for Tg(*ubi*:loxP-DsRed-STOP-loxP-dn-xBrg1); tamoxifen (4-HT) was applied at 3 days before injury. (**d**,**e**) AFOG staining revealed accumulated fibrin and fibrosis in Tg(*myl7*:CreER; *ubi*:DsRed-dn-xBrg1) transgenic hearts (**e**) compared with control Tg(*ubi*:DsRed-dn-xBrg1) transgenic hearts (**d**) at 30 d.p.a. (**f**,**g**) MF20 staining showed compromised myocardial regeneration in Tg(*myl7*:CreER; *ubi*:DsRed-dn-xBrg1) transgenic hearts (**g**) compared with control Tg(*ubi*:DsRed-dn-xBrg1) transgenic hearts (**f**) at 30 d.p.a. 4/4, all 4 hearts analysed showed the same phenotype. Scale bars, 100 μm.

Since Brg1 is induced in injured cardiomyocyte (Fig. 2h) and is essential for cardiomyocyte proliferation (Fig. 3e–g and Supplementary Fig. 8d–f), we tested whether the Brg1-cdkn axis acted in cardiomyocytes during heart regeneration. RNAscope *in situ* hybridization analysis revealed that both *cdkn1a* (Fig. 5a,b,i,j) and *cdkn1c* (Fig. 5e,f,m,n) were very lowly expressed in wild-type heart with or without injury, but both *cdkn1a* and *cdkn1c* was markedly induced in Tg(*hsp70*:dn-xBrg1) transgenic hearts (Fig. 5d,h,l,p) compared with wild-type sibling hearts (Fig. 5c,g,k,o). Interestingly, co-staining with MF20 showed that both *cdkn1a* and *cdkn1c* were enriched in the myocardium (Fig. 5l,p). Furthermore, by generating Tg(*myl7*:CreER;*ubi*:loxP-DsRed-STOP-loxP-dn-xBrg1) transgenic zebrafish, tamoxifen-induced myocardial-specific inhibition of Brg1 resulted in decreased PCNA^+^/Mef2C^+^ proliferating cardiomyocytes at 7 d.p.a. (Fig. 6a–c), as well as increased cardiac fibrosis (Fig. 6d,e) and compromised myocardial regeneration at 30 d.p.a. (Fig. 6f,g). Consistently, we also found that both *cdkn1a* and *cdkn1c* were upregulated in the MF20^+^ myocardium in Tg(*myl7*:CreER;*ubi*:loxP-DsRed-STOP-loxP-dn-xBrg1) transgenic hearts (Supplementary Fig. 10j,l,n,p) compared with Tg(*ubi*:loxP-DsRed-STOP-loxP-dn-xBrg1) control hearts (Supplementary Fig. 10i,k,m,o) after 4-HT induction by RNAscope. Together, these data support our hypothesis that Brg1 acts to suppress *cdkn1a* and *cdkn1c* in the myocardium to regulate heart regeneration in zebrafish.

**Figure 7:**
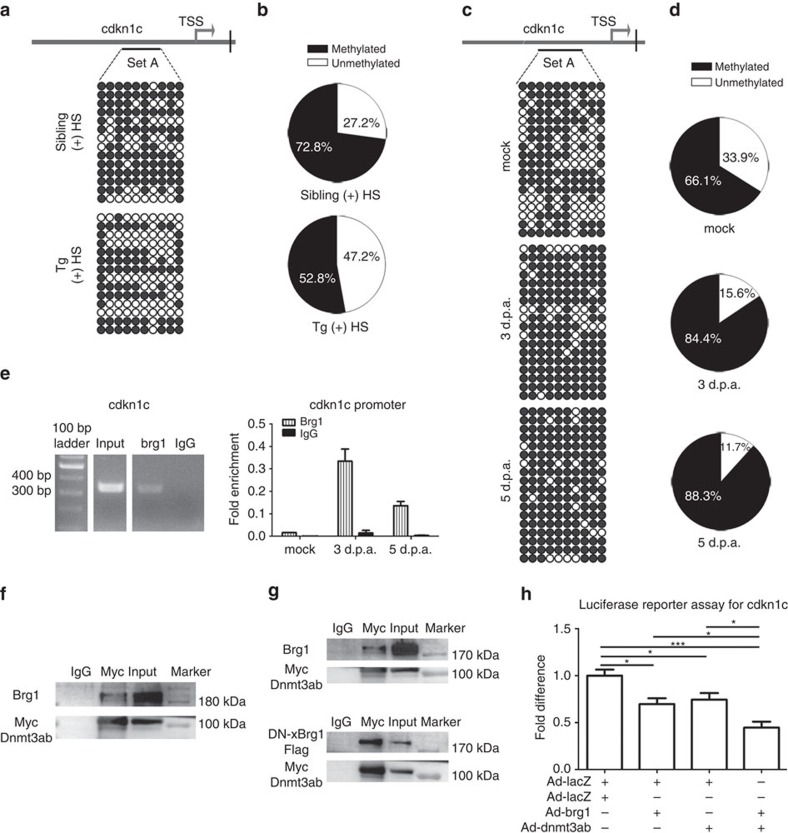
Brg1 represses *cdkn1c* expression by increasing the level of DNA methylation in its promoter region. (**a**) Methylation patterns of 10 individual CpG sites in the *cdkn1c* promoter of Tg(*hsp70*:dn-xBrg1) and wild-type sibling hearts after daily heat shock from 5 to 14 d.p.a. Upper panel, schematic of 10 CpG island sites (set A) of the *cdkn1c* promoter region and transcription start site (TSS); lower panels, *cdkn1c* methylation patterns of wild-type sibling (sibling) and dn-xBrg1 transgenic (tg) hearts, with open circles for ‘unmethylated’ and filled circles for ‘methylated’ CpG islands. Methylated DNA sequences were obtained by bisulfite sequencing. Note decreased methylation of *ckkn1c* promoter in dn-xBrg1 transgenic hearts (**b**). (**c**) *cdkn1c* promoter methylation of 10 individual CpG sites (set A) of mock, 3 d.p.a. and 5 d.p.a. wild-type hearts. The percentages of unmethylated (white) and methylated (black) DNA from **a** and **b** are shown in **b** and **d**. (**e**) Left panel, ChIP assays with anti-Brg1 antibody. Right panel, quantitation of Brg1 immunoprecipitated *cdkn1c* promoter in wild-type mock, 3 d.p.a. and 5 d.p.a. hearts. Data are presented as Brg1 enrichment relative to control IgG. The 335 bp DNA fragment within the *cdkn1c* promoter region (−1,625 to −1,290 bp) was amplified from immunoprecipitated DNA of mock, 3 d.p.a. and 5 d.p.a. hearts by anti-Brg1 antibody or control IgG. (**f**) Immunoprecipitation by anti-Myc antibody in 293T cells over-expressing Brg1 and Myc-tagged Dnmt3ab. (**g**) Upper panel, immunoprecipitation of Brg1 and Myc-Dnmt3ab by Myc antibody or control IgG antibody in H9C2 cells over-expressing Brg1 and Myc-tagged Dnmt3ab. Lower panel, immunoprecipitation of dn-xBrg1-Flag and Myc-Dnmt3ab by Myc antibody or IgG antibody in H9C2 cells over-expressing dn-xBrg1-Flag and Myc-Dnmt3ab. (**h**) Luciferase reporter assays showed that over-expression of zebrafish *brg1* and *dnmt3ab* synergistically suppressed the transcription of *cdkn1c* in 293T cells. 293T cells were transfected/infected with the indicated adenoviral constructs and luciferase reporter constructs, and those cells were then collected and measured for luciferase activity at 24 h after transfection/infection. Equal amounts of adenovirus were used for each group. Firefly luciferase activity was normalized by *Renilla* luciferase activity (*P<0.05, ***P<0.001; data are mean±s.e.m.; one-way analysis of variance followed by Bonferroni’s multiple comparison test).

We then asked how Brg1 regulates the transcriptional activation of CDK inhibitors such as *cdkn1c* during regeneration. Since DNA methylation is an important mechanism for regulating gene expression, we determined the DNA methylation pattern of the promoters of *cdkn1c*, *meis1a* and *tgfb1a* by performing bisulfate sequencing of 8–10 individual CpG sites. Bisulfate sequencing showed less methylation in these promoters of Tg(*hsp70*:dn-xBrg1) transgenic hearts than that in wild-type sibling hearts (Fig. 7a,b and Supplementary Fig. 11). Furthermore, methylation of the *cdkn1c* promoter increased in injured hearts at 3 and 5 d.p.a. while *brg1* was reciprocally induced compared with mock controls (Fig. 7c,d), confirming a potential endogenous role of Brg1 in repressing the expression of *cdkn1c*. To investigate the mechanism underlying the effects of Brg1 inhibition on *cdkn1c* promoter methylation, we performed chromatin immunoprecipitation (ChIP) and quantitative ChIP assays, and found that Brg1 bound with the *cdkn1c* promoter region, which was demethylated after inhibition of Brg1 during cardiac regeneration (Fig. 7a,e). Consistent with Brg1-cdkn1a/1c function in the myocardium, we found that *cdkn1c* promoter was hypomethylated in Tg(*myl7*:CreER;*ubi*:loxP-DsRed-STOP-loxP-dn-xBrg1) transgenic hearts compared with Tg(*ubi*:loxP-DsRed-STOP-loxP-dn-xBrg1) control hearts after 4-HT induction by bisulfite sequencing (Supplementary Fig. 12a); and that myc-tagged dn-Brg1 directly bound to the *cdkn1c* promoter in Tg(*myl7*:CreER;*ubi*:loxP-DsRed-STOP-loxP-dn-xBrg1) transgenic hearts by ChIP assay (Supplementary Fig. 12d).

Methyltransferases are known to maintain the patterns of methylated cytosine residues in the mammalian genome and are the key molecules in regulating the level of DNA methylation. Seven DNA methyltransferases were annotated in the zebrafish genome website (Zv9.0). By RT–PCR, we found that *dnmt1*, *dnmt3aa* and *dnmt3ab* were expressed in adult zebrafish heart, suggesting that both *de novo* and maintenance DNA methylation might occur during heart regeneration. By over-expressing Brg1 and Dnmt3ab in 293T cells, or H9C2 cardiac cells, we demonstrated that either Brg1 or dn-xBrg1 and Dnmt3ab form a protein complex by co-immunoprecipitation experiments (Fig. 7f,g). These data are consistent with previous studies in cancer cells^38^ and hypertrophic hearts^39^. An additional reporter system was further used to examine whether this interaction affected the *cdkn1c* promoter activity. Indeed, over-expression of either *brg1* or *dnmt3ab* inhibited the *cdkn1c* promoter activity, which was synergistically enhanced by co-expression of *brg1* and *dnmt3ab* in 293T cells (Fig. 7h) or P4 rat neonatal cardiomyocytes (Supplementary Fig. 12c). Furthermore, we also found that *baf60c* increased while *dnmt3ab* decreased in dn-xBrg1 transgenic hearts compared with their sibling hearts at 14 d.p.a. (Supplementary Fig. 13). These data suggest a feedback effect of SWI/SNF complex on baf60c, as well as synergistic interaction of Brg1 and Dnmt3ab in regulating DNA methylation of cdkn inhibitors on heart regeneration by over-expression of dn-xBrg1. Importantly, *dnmt3ab* was induced from 3 to 14 d.p.a. and peaked at 7 d.p.a. compared with that at mock hearts (Supplementary Fig. 14a), and nanoparticle-mediated *dnmt3ab* siRNA decreased BrdU^+^/Mef2C^+^ proliferating cardiomyocytes (Supplementary Fig. 14b–d), supporting the role of *dnmt3ab* during heart regeneration. We have previously reported methodology that nanoparticle-delivered siRNA efficiently inhibits targeted gene expression in adult zebrafish hearts^40^. Together, our data support the notion that Brg1 and Dnmt3ab form a protein complex in 293T cells and H9C2 cardiomyocytes, and this complex might be utilized to increase DNA methylation of *cdkn1c* promoter and so repressing its transcription for promoting zebrafish heart regeneration.

### Cdkn1a/1c mediate effects on myocardial proliferation

**Figure 8:**
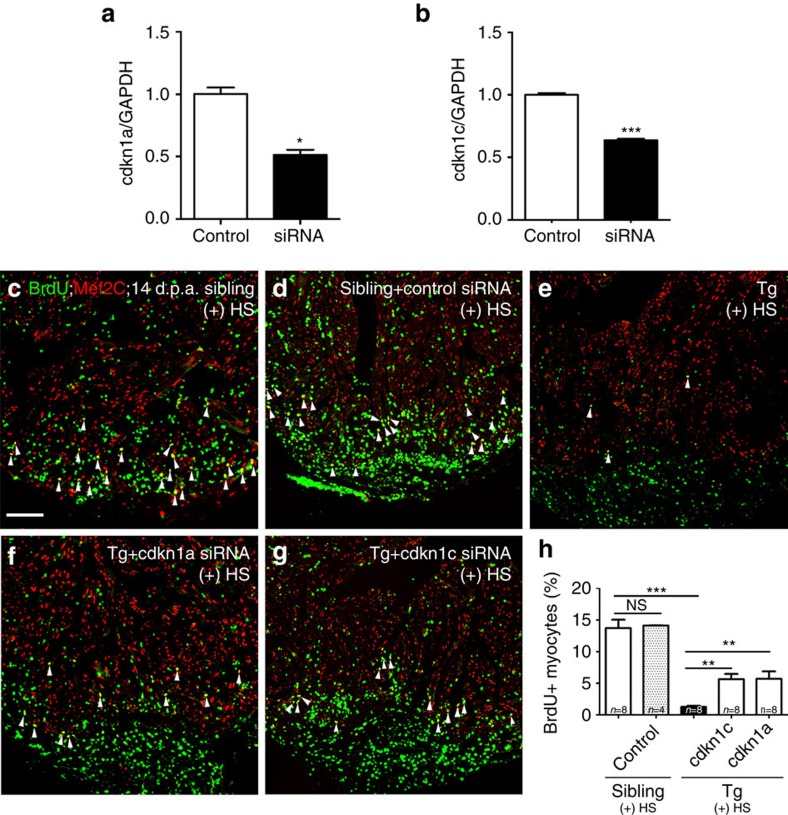
siRNA knockdown of either *cdkn1a* or *cdkn1c* partially rescues proliferating cardiomyocytes in the Tg(*hsp70*: dn-xBrg1) heart. (**a**,**b**) Quantitative PCR showed that nanoparticle-encapsulated siRNA efficiently decreased the RNA levels of *cdkn1a* and *cdkn1c* in wild-type hearts at 2 d.p.a., into which control and *cdkn1a* (**a**) or *cdkn1c* (**b**) siRNA were injected at 1 d.p.a. The RNA level was normalized to GAPDH (*P<0.05, ***P<0.001; data presented are mean±s.e.m.; paired Student’s *t*-test). (**c**–**h**) Ventricular apex amputation was performed in wild-type siblings and Tg(*hsp70*:dn-xBrg1) zebrafish, followed by heat shock treatment for 30 min daily from 5 to 14 d.p.a. The Mef2C^+^/BrdU^+^ double-positive cardiomyocytes were comparable in control siRNA-injected (**d**) and uninjected (**c**) hearts. Either encapsulated *cdkn1a* (**f**) or *cdkn1c* (**g**) siRNA partially rescued the ratio of Mef2C^+^/BrdU^+^ double-positive cardiomyocytes in Tg(*hsp70*:dn-xBrg1) hearts compared with those in uninjected control transgenic hearts (**e**). Scale bar, 100 μm. (**h**) Statistics of **c**–**g** (**P<0.01, ***P<0.001; data are mean±s.e.m.; one-way analysis of variance followed by Bonferroni’s multiple comparison test). The number (*n*) of hearts analysed in each group is indicated in each bar.

We then asked whether CDK inhibitors functionally act downstream from Brg1 during heart regeneration. Using nanoparticle-mediated siRNA knockdown method^40^, we were able to decrease the expression of *cdkn1a* and *cdkn1c* in 2 d.p.a. hearts at 24 h after siRNA injection (Fig. 8a,b). We found comparable BrdU^+^/Mef2C^+^ proliferating cardiomyocytes in wild-type sibling hearts without (Fig. 8c) or with control siRNA injection (Fig. 8d), showing that siRNA injections daily from 5 to 14 d.p.a. had no effect on injury-induced cardiomyocyte proliferation (Fig. 8h). Importantly, we found more BrdU^+^/Mef2C^+^ proliferating cardiomyocytes in Tg(*hsp70*:dn-xBrg1) hearts with either *cdkn1a* (Fig. 8f) or *cdkn1c* siRNA (Fig. 8g) than in control *dn-xBrg1* transgenic hearts (Fig. 8e,h). Another independent siRNA for either *cdkn1a* or *cdkn1c* was also able to rescue the proliferation index in Tg(*hsp70*:dn-xBrg1) transgenic hearts (Supplementary Fig. 15a–f). However, siRNA knockdown of either *cdkn1a* or *cdkn1c* had little or no effect on BrdU^+^/Mef2C^+^ proliferating cardiomyocytes in wild-type sibling hearts at 14 d.p.a. (Supplementary Fig. 16), consistent with very low levels of these genes in wild-type hearts after ventricle resection (Fig. 5). These data further support the notion that Brg1 promotes heart regeneration by repressing CDK inhibitors such as *cdkn1a* and *cdkn1c*.

## Discussion

Brg1 plays an essential role in embryonic development such as in zygote genome activation^14^, erythropoiesis^15^ and the development of T cells^41^,^42^, heart^16^,^17^ and neurons^18^,^19^. However, its function in adult heart regeneration has not been addressed. Here we showed that Brg1 was activated during zebrafish heart regeneration; either global or myocardial-specific over-expression of *dn-xBrg1* interfered with myocardial proliferation and regeneration; and mechanistically, Brg1 promoted regeneration by suppressing the CDK inhibitors *cdkn1c*. Although Brg1 was broadly expressed in cardiomyocytes, endothelial/endocardial cells, epicardium and inflammatory cells (macrophages and neutrophils), myocardial-specific enrichment and function of Brg1 and *cdkn1c* suggest that the Brg1-*cdkn1c* axis acts in the myocardium to regulate cardiomyocyte proliferation and regeneration. Future studies are warranted to parse Brg1 function in the endocardium, epicardium and inflammatory leukocytes in adult heart regeneration.

Previous studies have shown that the components of the SWI/SNF complex (also called the BAF complex) are differentially expressed in embryonic stem cells, neuronal progenitors and differentiated neurons^43^, suggesting the existence of cell- or tissue-specific SWI/SNF complexes. Embryonic stem cell BAF contains Brg1, BAF250a, BAF60a/b, BAF155, BAF57, BAF47, BAF53a, BAF45a/d and SS18 (ref. 13). Neuronal progenitor BAF consists of Brg1 or Brm, BAF250a/b, two BAF155 homodimers or BAF155/170 heterodimers, BAF53a, BAF45a, BAF60a/c, BAF47, BAF45a/d and SS18, which are essential for maintaining the stem cell state^44^. After neuronal progenitor differentiation into neurons, the BAF complex changes from BAF53a to 53b, SS18 to CREST, and BAF45a/d to 45b/c to form neuronal BAF^45^. It remains unclear whether there is a cardiac regenerative BAF complex. During zebrafish heart regeneration, we found that *brg1*, *baf60c* and *baf180* were induced on heart sections. They had no or little expression in control hearts and were induced from 1 d.p.a. with peak expression around 7–14 d.p.a., and then declined from 14 to 30 d.p.a. for *brg1* and *baf60c* while declined from 21 to 30 d.p.a. for *baf180*. Overall, *brg1* and *baf60c* mRNA are more abundant while *baf180* is less expressed during heart regeneration. Therefore, we propose that a similar cardiac-regenerative BAF complex might contain at least *brg1*, *baf60c* and *baf180* during heart regeneration in zebrafish. However, this hypothesis remains to be tested in future studies.

Previous studies by Field and colleagues have shown that either deletion of a CDK inhibitor or activation of a CDK has limited effects on promoting mammalian cardiomyocyte proliferation^2^,^12^,^27^,^28^,^29^. Our data support the notion that an increase of CDK inhibitors and related meis genes occurs after inhibition of Brg1 during heart regeneration in zebrafish; this was partly supported by RNA-seq analysis of Tg(*hsp70*:dn-xBrg1) and wild-type sibling hearts after daily heat shock from 5 to 14 d.p.a., as well as being further confirmed by quantitative RT–PCR and its myocardial enrichment by RNAscope *in situ* hybridization analysis. During regeneration, *cdkn1c* was downregulated in injured hearts at 3 and 5 d.p.a., while *brg1* was reciprocally upregulated, suggesting that Brg1 normally represses *cdkn1c* expression during this process, consistent with previous reports that cell-cycle-dependent kinase inhibitors are downstream of Brg1 in cardiac development^17^, mammalian neural crest cell development^24^, bulge stem cells during tissue regeneration^26^ and adult neural stem cells maintenance^46^, as well as Brg1 directly binds to the *cdkn1c* promoter as predicted by ChIP-seq analysis^47^. Furthermore, our data showed that Brg1 directly bound to the promoter of *cdkn1c* by ChIP assay, repressed the *cdkn1c*-luciferase reporter, and siRNA knockdown of either *cdkn1a* or *cdkn1c* partially rescued the blunted myocardial proliferation with transgenic over-expression of dn-*xBrg1*. Taken together, we have shown, for the first time, that Brg1 promotes adult cardiomyocyte proliferation by repressing CDK inhibitors, specifically by direct repression of *cdkn1c* during heart regeneration.

It has been shown that Brg1 interacts directly or indirectly with other transcription factors or epigenetic components^17^,^24^,^25^,^26^,^38^,^48^, and SWI/SNF chromatin-remodelling factors can induce changes in DNA methylation to regulate gene expression^49^. We then hypothesized that Brg1 might interact with other transcription and/or epigenetic factors to repress the transcription of *cdkn1c*. DNA methyltransferases are known to catalyse the reaction of transferring the methyl group to DNA from *S*-adenosyl methionine. Dnmt3a and Dnmt3b are *de novo* DNA methyltransferases that normally act as transcriptional repressors by DNA methylation or transcriptional co-repressors^38^,^50^,^51^,^52^,^53^,^54^,^55^,^56^. Here we showed that inhibition of Brg1 led to a decreased level of DNA methylation in the promoters of *cdkn1c*, *meis1a* and *tgfb1a*, and increased the expression of *cdkn1c* accordingly. Importantly, the level of DNA methylation in the *cdkn1c* promoter increased after ventricular resection at 3 and 5 d.p.a., which is consistent with the repression of *cdkn1c* during normal regeneration. Indeed, RT–PCR showed that zebrafish *dnmt3ab* was induced during heart regeneration and it is required for myocardial proliferation, and co-immunoprecipitation analysis showed that it directly interacted with Brg1 in 293T cells and H9C2 cardiomyocytes, consistent with the previous report that Brg1 and Dnmt3a interact in cancer cells^38^ and in hypertrophic cardiomyocytes^39^. Our data reveal that *dnmt1*, *dnmt3aa* and *dnmt3ab* are expressed in adult zebrafish heart, and their respective role in heart regeneration need to be addressed in the future. Together, Brg1 suppresses expression of *cdkn1c* and possible other CDK inhibitors, at least, partly through its interacting with Dnmt3ab to increase the level of DNA methylation in the *cdkn1c* promoter, leading to an automatic regenerative capacity in the heart of adult zebrafish. Therefore, conditional activation of the BAF complex and related signalling pathways might shed light on improving mammalian myocardial regeneration.

## Methods

### Zebrafish lines

Zebrafish were raised and handled according to a zebrafish protocol (IMM-XiongJW-3) approved by the Institutional Animal Care and Use Committee at Peking University, which is fully accredited by AAALAC International. Tg(*hsp70*:dn-xBrg1), Tg(*myl7*:cypher-EGFP), Tg(*myl7*:CreER), Tg(*ubi*:loxP-DsRed-STOP-loxP-Brg1) and Tg(*ubi*:loxP-DsRed-STOP-loxP-dn-xBrg1) zebrafish lines were generated by using Tol2-based transgenesis^57^. The dominant-negative *Xenopus Brg1* (*dn-xBrg1*) plasmid clone was kindly provided by Dr Kristen L. Kroll (Washington University at St Louis)^19^,^58^, and the Tg(*myl7*:CreER) and Tg(*ubi*:loxP-DsRed-STOP-loxP-EGFP) plasmid clones were kindly provided by C Geoffrey Burns (Massachusetts General Hospital, Boston, MA, USA)^59^. Tg(*coronin1a*:EGFP)^60^ and Tg(*flk1*:nucEGFP)^61^ lines were provided by Dr Zilong Wen (Hong Kong University of Science and Technology, Hong Kong, China) and Dr Feng Liu (Institute of Zoology, Chinese Academy of Sciences, Beijing, China); Tg(*gata4*:EGFP) line^62^ was provided by Dr Todd Evans (Weill Cornell Medical College, New York, USA); and Tg*(tcf21*:DsRed) line^63^ was provided by C. Geoffrey Burns (Massachusetts General Hospital). Heterozygous transgenic zebrafish and their wild-type siblings were used for all experiments.

For heat shock experiments, we crossed heterozygous Tg(*hsp70*:dn-xBrg1) with wild-type TL zebrafish, and so expected to have 50% heterozygous transgenic fish and 50% wild-type siblings. Heterozygous Tg(*hsp70*:dn-xBrg1) transgenic and wild-type sibling adult zebrafish received a daily heat shock in 37 °C water for 30 min from 5 to either 14 or 30 d.p.a. Each cycle of heat shock was carried out by transferring zebrafish to system water at 31 °C, accompanying with gradually increasing temperature from 31 to 37 °C for about 10 min and then remaining at 37 °C for another 20 min. To induce the Cre recombination in adult zebrafish, Tg(*myl7*:CreER;*ubi*:loxP-DsRed-STOP-loxP-dn-xBrg1) or control Tg(*ubi*:loxP-DsRed-STOP-loxP-dn-xBrg1) transgenic zebrafish were bathed in 5 μM tamoxifen (Sigma, St Louis, MO) for 24 h, which was made from a 10 mM stock solution dissolved in 100% ethanol at room temperature. Zebrafish were treated with tamoxifen at a density of 3–4 per 150 ml of water, and then returned to circulating zebrafish system water. Ventricular resections were performed at 3 days after tamoxifen treatment. Zebrafish were confirmed for their genotyping and randomly picked for all experiments.

### Adult zebrafish heart resection

The ventricular resection was performed according to a well-established procedure^5^,^34^. Briefly, adult zebrafish were anaesthetized with tricaine and the pericardial sac was exposed by removing surface scales and a small piece of skin. The apex of the ventricle was gently pulled up and removed with Vannas scissors. The zebrafish was then placed back into a water tank, and water was puffed over the gills with a plastic pipette until it breathed and swam regularly. The surface opening sealed automatically within a few days. In particular, we crossed heterozygous Tg(*hsp70*:dn-xBrg1) with wild-type TL zebrafish, and so expected to have 50% heterozygous transgenic fish and 50% wild-type siblings for performing ventricular resections.

### Construction and sequencing of high-throughput RNA-seq libraries

Total RNA was isolated using an RNeasy Mini kit (QIAGEN). After confirming the quality and integrity of RNA on agarose gels, we used ∼1 μg of total RNA to construct the RNA-seq libraries by applying a TruSeq RNA Sample Prep kit (Illumina, San Diego, CA). We carried out the RNA-seq using Illumina HiSeq 2500 to generate ∼4 million 100 bp pair-end reads for each sample. Low-quality reads and sequencing adapters were removed from the raw sequencing data, and the clean reads were mapped onto the zebrafish transcriptome (danRer7) using Tophat^64^. The expression level of each gene was calculated using Cufflinks, and the genes differentially expressed between samples were calculated using Cuffdiff^64^. Genes with >2-fold difference between the two groups were selected for further analyses.

### siRNA delivery into adult zebrafish heart

siRNAs were encapsulated in polyethylene glycol–polylactic acid nanoparticles using a double emulsion-solvent evaporation technique and then injected into the pericardial sac^40^,^65^,^66^. Briefly, zebrafish were allowed to recover for 1 day after ventricular resection. To evaluate the effect of siRNA on its target gene expression, the hearts were collected at 2 d.p.a., and total RNA was isolated to assess the expression of the respective genes by quantitative RT–PCR. To evaluate the effect of genes on cardiomyocyte proliferation, 50 μl polyethylene glycol–polylactic acid nanoparticle-encapsulated siRNAs was injected first, and ∼1 h later, 50 μl 2.5 mg ml^−1^ BrdU (B5002; Sigma) was injected into the thoracic cavity daily from 7 to 14 d.p.a. The hearts at 14 d.p.a. were collected for subsequent experiments. siRNA sequences for *cdkn1a*, *cdkn1c* and *dnmt3ab* are shown in Supplementary Table 2.

### mRNA or protein detection assays and AFOG staining

*In situ* hybridization and AFOG staining were performed on paraffin sections^34^. Adult zebrafish hearts were fixed in 4% paraformaldehyde at room temperature for 2 h, dehydrated and then embedded in paraffin and sectioned at 5 μm. Zebrafish *brg1* cDNA was cloned from an embryonic cDNA library, of which primer sequences are shown as brg1-F and brg1-R in Supplementary Table 1. Digoxigenin-labelled *brg1* probes were synthesized using T7 RNA polymerase (Roche).

For immunofluorescence staining, adult zebrafish hearts were fixed in 4% paraformaldehyde at room temperature for 2 h, dehydrated and then embedded in paraffin and sectioned at 5 μm. The sections were dewaxed in xylene, rehydrated with a series of ethanol and then washed in PBS. To repair the antigen, the citric acid buffer (CW0128S; CWBIO) and the microwave treatment were used. After washing in water and PBS, the sections were blocked in 10% FBS in PBT (1% tween 20 in PBS), and then incubated with primary antibodies (1:50 diluted in PBT containing 10% FBS) overnight at 4 °C. The primary antibodies used for immunofluorescence were anti-BrdU (B8434; Sigma), anti-Mef2c (sc-313; Santa Cruz), anti-GFP (A-11122; Invitrogen), anti-PCNA (18-0110; Invitrogen), anti-GFP (BE2001; EASYBIO), anti-RFP (BE2023; EASYBIO), anti-myosin heavy-chain monoclonal antibody (hybridoma product MF20; Developmental Studies Hybridoma Bank, Iowa City, IA) and The Brg1 JI antibody, which was raised against a glutathione *S*-transferase–BRG1 fusion protein (human BRG1 amino acids 1,086–1,307)^30^,^67^. The primary antibodies were then washed and sections were incubated with secondary antibodies for 2 h at room temperature. Secondary antibodies (1:100 diluted in PBT) were Alexa Fluor 488 goat anti-mouse IgG (A21121; Invitrogen), Alexa Fluor 488 goat anti-rabbit IgG (A11034; Invitrogen), Alexa Fluor 555 goat anti-mouse IgG (A21424; Invitrogen) and Alexa Fluor 555 goat anti-rabbit IgG (A21428; Invitrogen).

RNAscope (Advanced Cell Diagnostics, Hayward, CA) was performed on 10 μm sections from freshly frozen hearts embedded in O.C.T. Compound (Embedding Medium for Frozen Tissue Specimens to ensure Optimal Cutting Temperature; SAKURA; 4583). Tissues were fixed in pre-chilled 10% neutral buffered formalin, followed by dehydration, then treated with Pretreat 1 for 10 min at room temperature. After Pretreat 1, slides were washed with water and incubated for 30 min at room temperature with Pretreat 4. Following Preteat 4, the RNAscope 2.0 HD Detection Kit Brown was applied for visualizing hybridization signals. Three injured and mock hearts were used for each RNAscope experiment. Immunostaining was performed with primary antibodies (1:50 diluted in PBT containing 10% FBS) incubated overnight at 4 °C.

RNA *in situ* hybridization, RNAscope *in situ* hybridization and AFOG staining were analysed and documented under a fluorescence microscope (DM5000B; Leica, Germany). Immunofluorescence images were captured on a confocal microscope (LSM510; Carl Zeiss, Germany). A Zeiss 700 confocal microscope was used for RNAscope with Immunostaining images. The BrdU^+^/Mef2C^+^, PCNA^+^/Mef2C^+^, Brg1^+^, MF20^+^/Brg1^+^, Flk1^+^/Brg1^+^ and Coronin1a^+^/Brg1^+^ were counted manually. Fluorescence intensity was quantitated using MBF Image J.

### RT–PCR analysis

Total RNA was isolated and purified using an RNeasy mini kit (74106; Qiagen). About 1 μg RNA was used for reverse transcription with a Prime Script RT Reagent kit (RR037A; TakaRa), and quantitative RT–PCR was performed using a SYBR Premix DimerEraser kit (RR091A; TakaRa). Primer sequences are listed in Supplementary Table 1.

### Chromatin immunoprecipitation

Chromatin was isolated from zebrafish hearts using Chromatin Prep Module (Catalogue# 26158; Thermo Scientific Pierce). ChIP assays were performed using Agarose ChIP Kit (Catalogue# 26156; Thermo Scientific Pierce)^34^. Chromatin was immunoprecipitated using anti-Brg1 (J1) antibody^30^, and Brg1-bound sequences were amplified with the respective gene primers. The primer sequences are listed in Supplementary Table 1. The original uncropped images of gels are shown in Supplementary Fig. 17.

### DNA methylation and immunoprecipitation

Zebrafish genomic DNA was extracted, purified and resuspended in 1 × TE buffer. For each sample, a total of 500 ng genomic DNA was treated using MethylCode bisulfite conversion kit (MECOV-50; Invitrogen). The CpG islands of the *cdkn1c* promoter region were primarily located between −1,586 and −1,354 bp. The bisulfite-treated DNA was subjected to PCR to amplify the *cdkn1c* promoter region. The primers were designed according to the website (http://www.urogene.org/methprimer/) and are listed in Supplementary Table 1. Alterations of promoter methylation were confirmed by Sanger sequencing. In addition, zebrafish *dnmt3ab* was isolated from an embryonic cDNA library and then subcloned into the pCDNA3.1 vector. Myc-tagged *dnmt3ab* and *brg1* were co-transfected into 293T cells (CRL-1573, American Type Culture Collection; ATCC), which were then collected for immunoprecipitation after 24 h (ref. 34). For immunoprecipitation, H9C2 cells (CRL-1446, ATCC) were infected with either Ad-*brg1*/Ad-Myc-*dnmt3ab* or Ad-*dn-xbrg1-*Flag/Ad-Myc *dnmt3ab*, and infected cells were then collected for immunoprecipitation after 24 h. The recombinant adenovirus was constructed and amplified by SinoGenoMax Co., Ltd, Beijing, China. The antibody for immunoprecipitation were anti-Myc (2276S; Cell Signaling Technology), anti-Flag (AP1013a, ABGENT) and anti-Brg1 (J1) as described previously^30^. The original uncropped images of blots are shown in Supplementary Fig. 17.

### Luciferase assays

The *cdkn1c* promoter (from −1,624 to −1192, bp) was cloned into the chromatinized pREP4 vector to form pREP4-cdkn1c-Luc. 293T cells (CRL-1573, ATCC) or primary cultured cardiomyocytes from neonatal P4 rats were transfected with pREP4-cdkn1c-Luc (495ng) and pREP4-renilla (5ng) by Lipofectamine 3000 (Invitrogen). At 20 h after transfection, the 293T cells were co-infected with Ad-*lacZ*, Ad-*brg1*, Ad-*dnmt3ab* or Ad-*brg1*/Ad-*dnmt3ab*, respectively. Luciferase assays were carried out 48 h after adenovirus infection^68^. Firefly luciferase activity was normalized by *Renilla* luciferase activity.

### Transmission electron microscopy

Tg(*hsp70*:dn-xBrg1) and wild-type sibling hearts were collected at 14 d.p.a. and fixed in 2% glutaraldehyde, 2% paraformaldehyde and 0.1 M PBS overnight at 4 °C. Subsequent embedding, ultra-thin section preparation and staining were performed by the Electron Microscopy Core Facility of Peking University. A TecnaiT20 (LaB6, 200KV) transmission electron microscope (FEI, Hillsboro, OR, USA) was used to image stained sections^33^. Three transgenic and wild-type sibling hearts were used for transmission electron microscopy.

### Statistical analysis

All statistics were calculated using Prism 5 Graphpad Software. The statistical significance between two groups was determined using paired Student’s *t*-test, with tow-tailed *P* value, and the data were reported as mean±s.e.m. Among three or more groups, one-way analysis of variance followed by Bonferroni’s multiple comparison test or Dunnett’s multiple comparison test was used for comparisons.

### Data availability

Data that support the findings of this study have been deposited in Gene Expression Omnibus with the accession code GSE81627. All other relevant data are available from the corresponding authors on reasonable request.

## Additional information

**How to cite this article:** Xiao, C. *et al*. Chromatin-remodelling factor Brg1 regulates myocardial proliferation and regeneration in zebrafish. *Nat. Commun.* **7,** 13787 doi: 10.1038/ncomms13787 (2016).

**Publisher's note:** Springer Nature remains neutral with regard to jurisdictional claims in published maps and institutional affiliations.

## Supplementary Material

Supplementary Informations Supplementary Figures and Supplementary Tables.

Supplementary Data 1 Down-regulated genes in dn-xBrg1 transgenic hearts.

Supplementary Data 2 Up-regulated genes in dn-xBrg1 transgenic hearts.
