# Supplementary material for: Chromatin-remodelling factor Brg1 regulates myocardial proliferation and regeneration in zebrafish
Source: Nat Commun. 2016 Dec 8;7:13787. doi: 10.1038/ncomms13787 (PMC5476829; doi:10.1038/ncomms13787)
Supplement: Supplementary Informations — Supplementary Figures and Supplementary Tables. [file ncomms13787-s1.pdf]

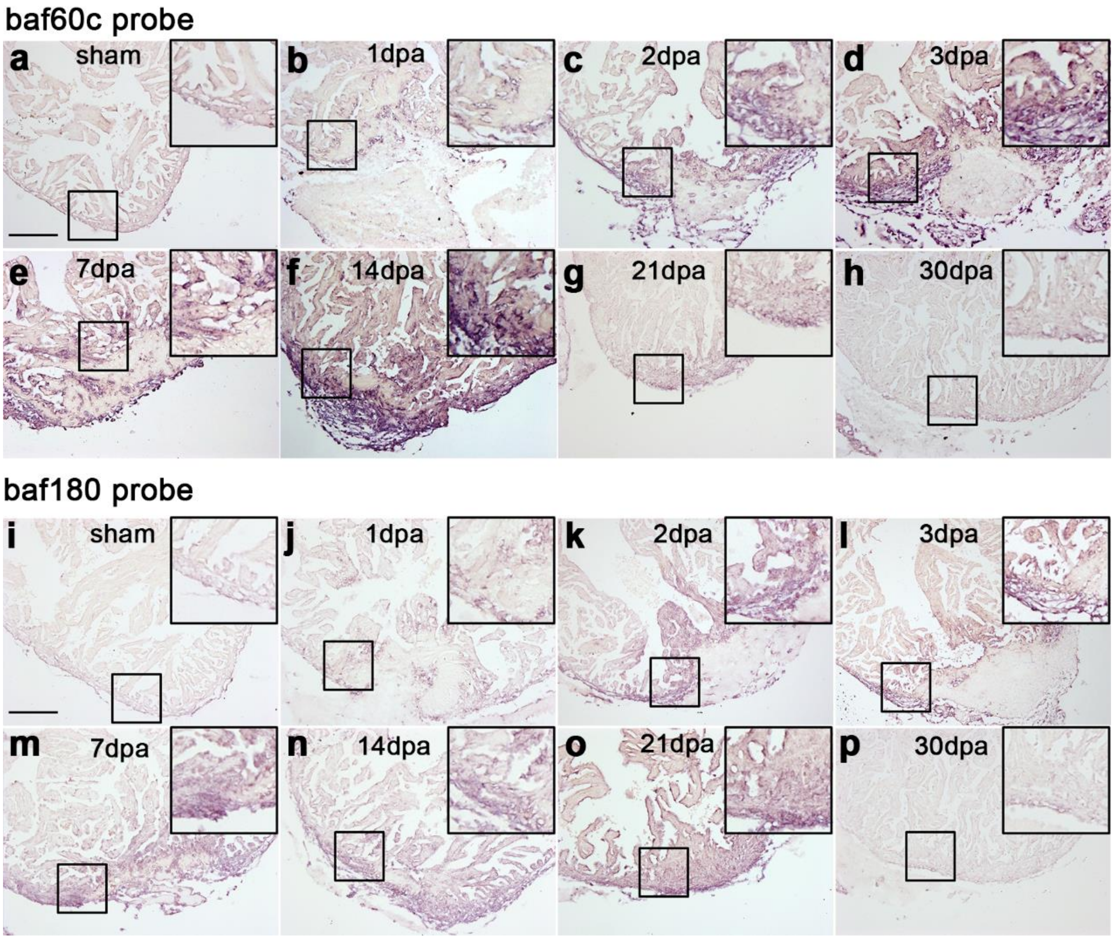

Supplementary Figure 1. *Baf60c* and *baf180* are induced during cardiac regeneration in zebrafish. RNA *in situ* hybridization was performed on paraffin sections from sham-operated adult hearts (a and i) and those with amputated ventricular apices from 1 to 30 dpa (b-h and j-p) with digoxigenin-labeled *baf60c* probe (a-h) or *baf180* probe (i-p). Higher-magnification images of areas in squares were shown in the upper-right corners of panels a-p. Scale bars, 100 μm. Representative data from 3 independent experiments (n=5 hearts).

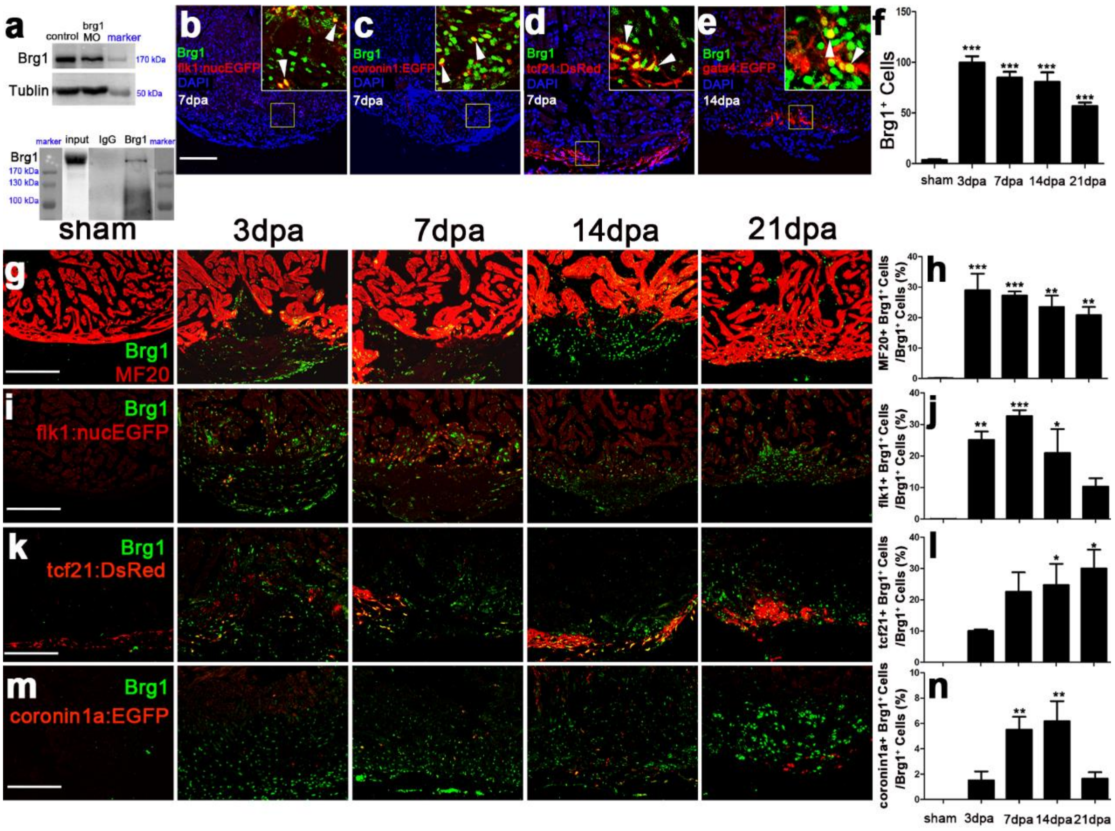

Supplementary Figure 2. Brg1 is activated in multiple types of cells during cardiac regeneration. (a) Upper panel, western blot with anti-Brg1 antibody showing that Brg1 decreased in *brg1* morphant embryos compared with control embryos at 48 hpf. Tubulin served as a loading control. Lower panel, Immunoprecipitation (IP) by anti-Brg1 antibody showing that Brg1 antibody was able to pull down the endogenous Brg1 of adult zebrafish hearts at 7 dpa. (b-e) Immunostaining of Brg1 and EGFP or DsRed on paraffin sections of injured hearts, in which the endocardium/endothelium were labeled by Tg(*flk1*:nucEGFP) at 7 dpa (b), macrophages and neutrophils by Tg(*coronin1a*:EGFP) at 7 dpa (c), the epicardium by Tg(*tcf21*:DsRes) at 7dpa (d), and the myocardium by Tg(*gata4*:EGFP) at 14 dpa (e). In the upper-right corners of panels b-e, higher magnification images show that Brg1 was located in the endocardium (b), macrophages/neutrophils (c), the epicardium (d), and the myocardium (e). (f) Quantification of Brg1-positive cells of sham and injured hearts from 3 to 21 dpa. (g-n) Immunostaining of Brg1 and MF20 (g), Brg1 and flk1:nucEGFP (i), Brg1 and tcf21:DsRed (k), as well as Brg1 and coronin1a:EGFP (m) of sham and injured hearts from 3 to 21 dpa. Quantification of Brg1^{+} cells co-expressing MF20 (h), Brg1^{+} cells co-expressing *flk1*:nucEGFP (j), Brg1^{+} cells co-expressing *tcf21*:DsRed (i), and Brg1^{+} cells co-expressing *coronin1a*:EGFP (n). Scale bars,100 μm. For all quantifications, data are mean ± s.e.m.; one-way ANOVA followed by Dunnett's Multiple Comparison Test, *p <0.05, **p <0.01, ***p <0.001.

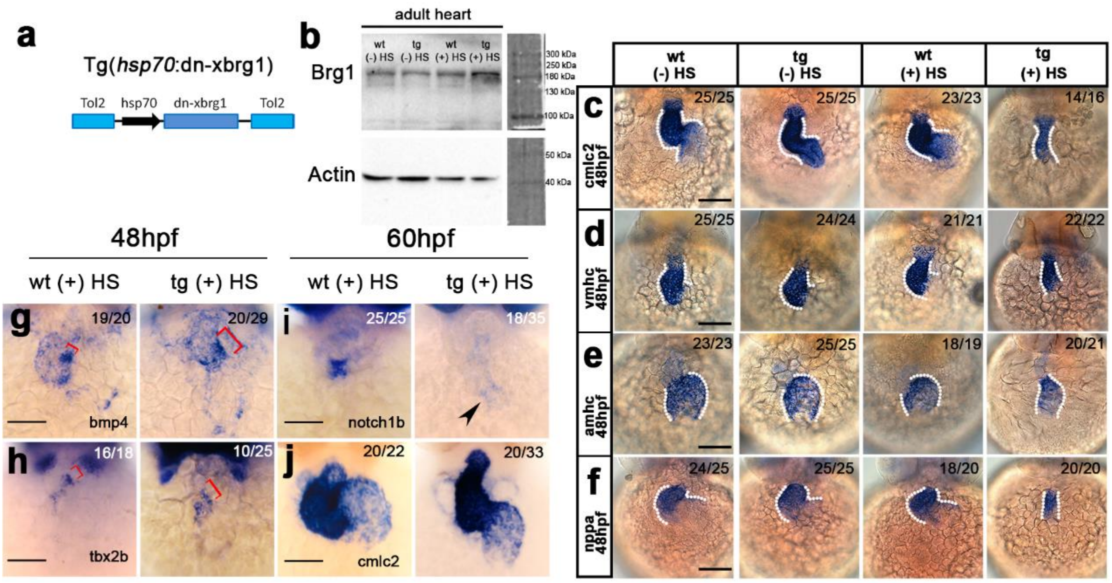

Supplementary Figure 3. Overexpression of *Xenopus* dominant-negative Brg1 (*dn-xBrg1*) inhibits *brg1* function in zebrafish. (a) Schematic of the Tol2-based construct of conditional expression of *dn-xBrg1* driven by the heat-shock promoter 70 (*hsp70*). (b) Western blots showing that heat-induced dn-xBrg1 protein increased in transgenic adult hearts (tg) compared with those in non-transgenic wild-type siblings (wt) with (+HS) or without heat shock (-HS), or transgenic adult hearts without heat shock (-HS). Alpha-actin served as a loading control. (-) HS, without heat shock; (+) HS, with heat shock. (c-f) Heart tube became stenotic and abnormal looping in tg embryos compared with wt sibling embryos at 48 hpf after heat shock (30 min each time at 5 hpf, 17 hpf, 29 hpf, and 41 hpf) or wt and tg embryos without heat shock. The heart tube was labeled by *in situ* hybridization with *cmlc2*, *vmhc*, *amhc*, or *nppa* probes. In situ hybridization showing that cardiac genes *bmp4*, *tbx2b*, and *notch1b* were abnormally expressed in tg embryos compared with wt sibling embryos after heat shock (30 min each time at 9 hpf, 33 hpf and 57 hpf) (g, h, i). Note the expanded *bmp4* (g) and *tbx2b* (h) domains and decreased expression of *notch1b* in the atrioventricular canal of tg embryos (i). (j) Heart tube, labeled by *cmlc2*, became stenotic but the atrium and ventricle were specified in tg embryos compared with wt sibling embryos at 60 hpf after heat shock. Red brackets indicate expression domains of atrioventricular canal markers (*bmp4* and *tbx2b*); black arrowheads point to decreased expression of *notch1b* in tg embryos; number of the right-upper corners showing the number of phenotypic embryos out of the total embryos analyzed. Scale bars, 100 µm.

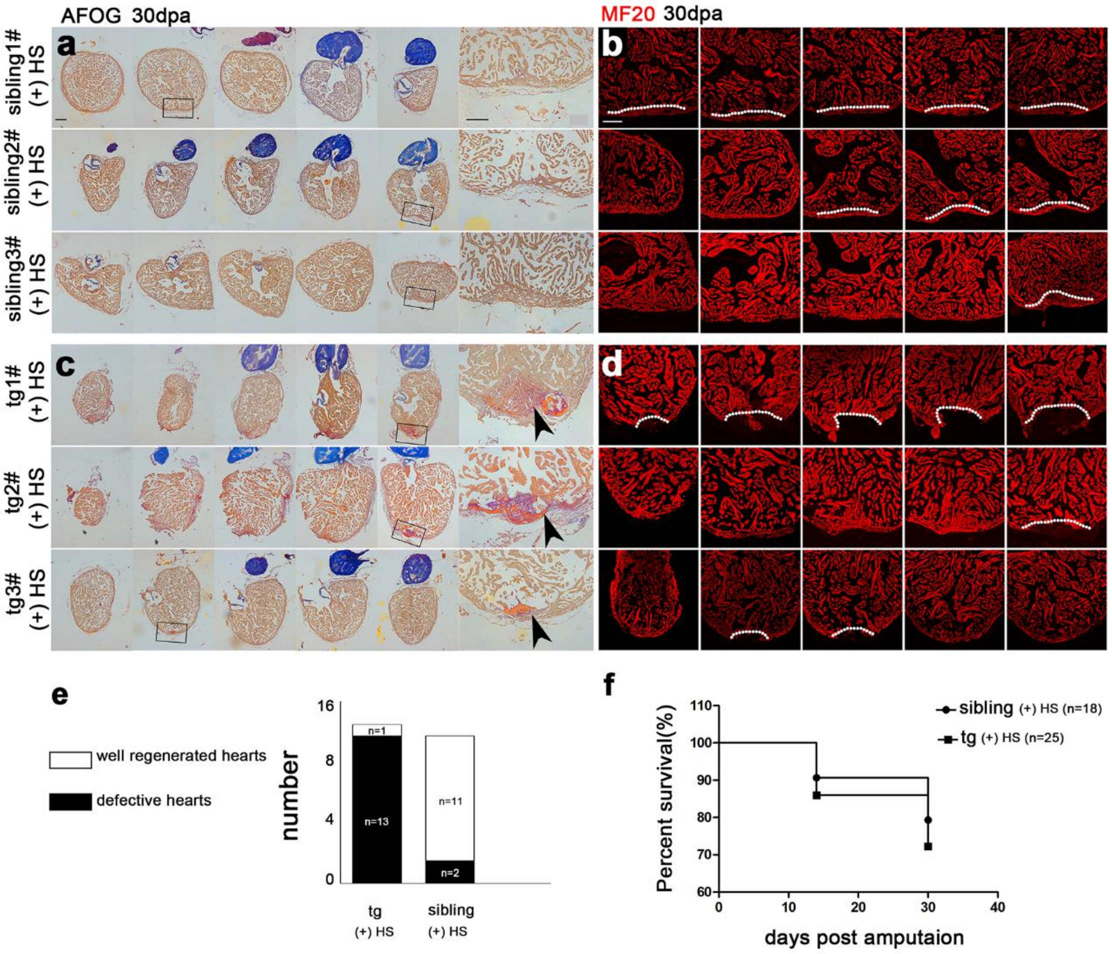

Supplementary Figure 4. Inhibition of *brg1* impairs cardiac regeneration. (a-d) Three wild-type (wt) sibling hearts (a-b) and 3 dn-xBrg1 transgenic (tg) hearts (c-d) at 30 dpa with heat shock treatment from 5dpa to 30dpa were subjected to serial sections, and half of sections were then used for AFOG staining (a, c) and half for MF20 immunofluorescence staining (b, d). Higher-magnification images of areas in squares were shown in the right side of panels a and c. Note cardiac fibrosis (black arrowheads) and compromised myocardial regeneration (dashed lines) in tg hearts (c, d) compared with perfect heart regeneration in wt sibling hearts (a, b). Scale bars, 100 µm. (e) Quantification of defective hearts; the number of well-regenerated hearts (white) or defective hearts (black) in each group was indicated in each bar (n=13 for sibling and n=14 for dn-xBrg1 transgenic total hearts). (f) Heat-induced lethality of wt siblings (n=18) and tg zebrafish (n=25) after heat shock at 14 dpa and 30 dpa.

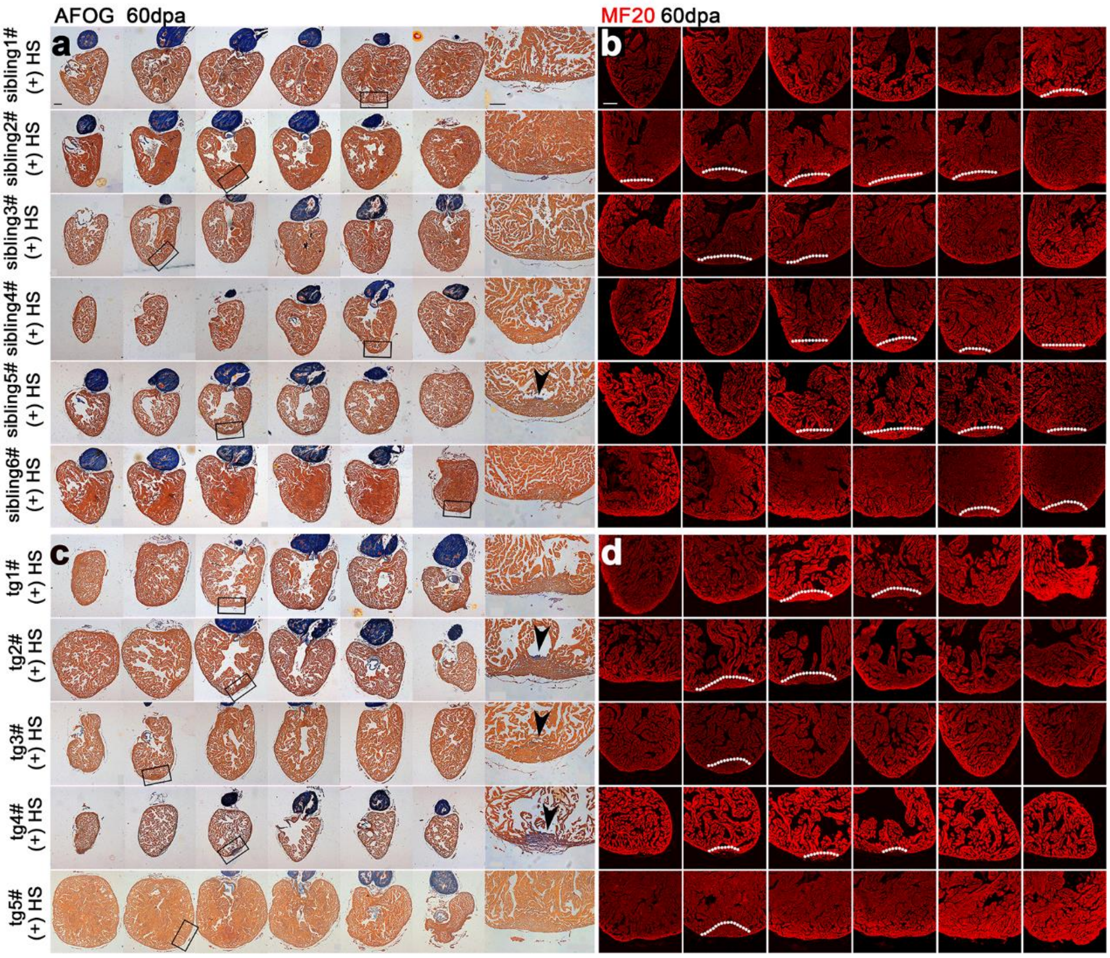

Supplementary Figure 5. Inhibition of Brg1 causes permanent defects in heart regeneration. Six wild-type (wt) sibling hearts (a-b) and 5 dn-xBrg1 transgenic (tg) hearts (c-d) at 60 dpa (heat shock treatment only from 5dpa to 30dpa) were subjected to serial sections, and half of sections were then used for AFOG staining (a, c) and half for MF20 immunofluorescence staining (b, d). Higher-magnification images of areas in squares were shown in the right side of panels a and c. Note that 3 of 5 tg hearts had fibrosis and compromised myocardial regeneration even although they had no heat-induced dn-xBrg1 proteins from 30 to 60 dpa. Arrowheads indicate fibrosis. Scale bars, 100 μm.

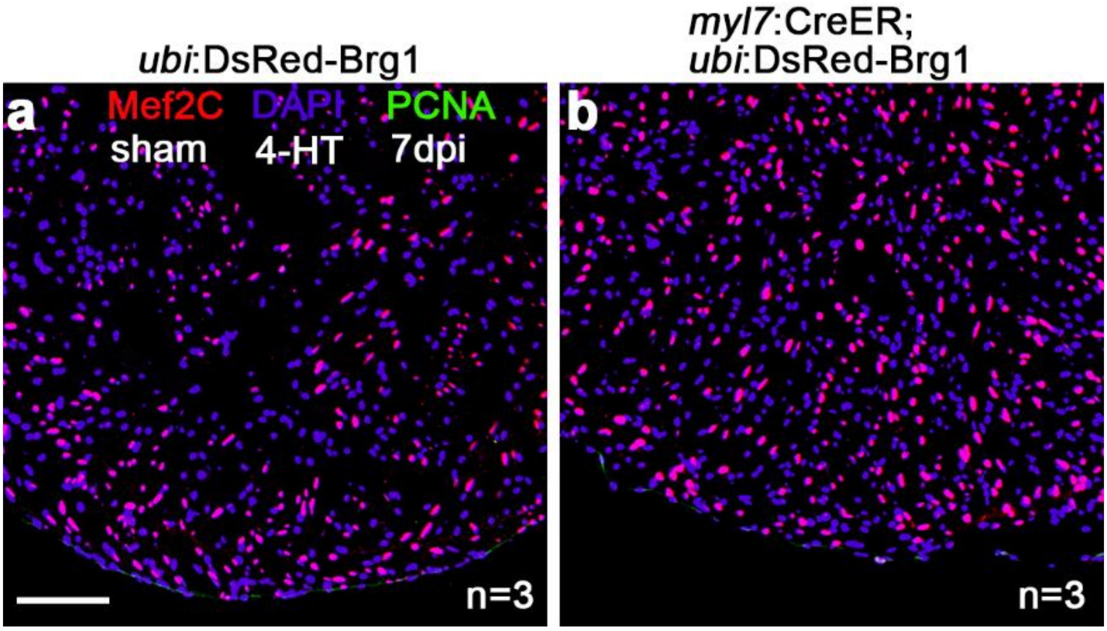

Supplementary Figure 6. Cardiomyocyte-specific overexpression of *brg1* is not sufficient to induce myocyte proliferation. PCNA^{+}/Mef2C^{+} proliferating cardiomyocytes were not increased in uninjured Tg(*myl7*:CreER; *ubi*:loxP-DsRed-STOP-loxP-Brg1) transgenic hearts (b) compared with uninjured control Tg(*ubi*:loxP-DsRed-STOP-loxP-Brg1) hearts (a). Hearts were induced by tamoxifen for 24hr, and 7 days post inducement paraffin heart sections were costained for PCNA (green), Mef2C (red) and DAPI (purple). n=3; Scale bar, 100μm.

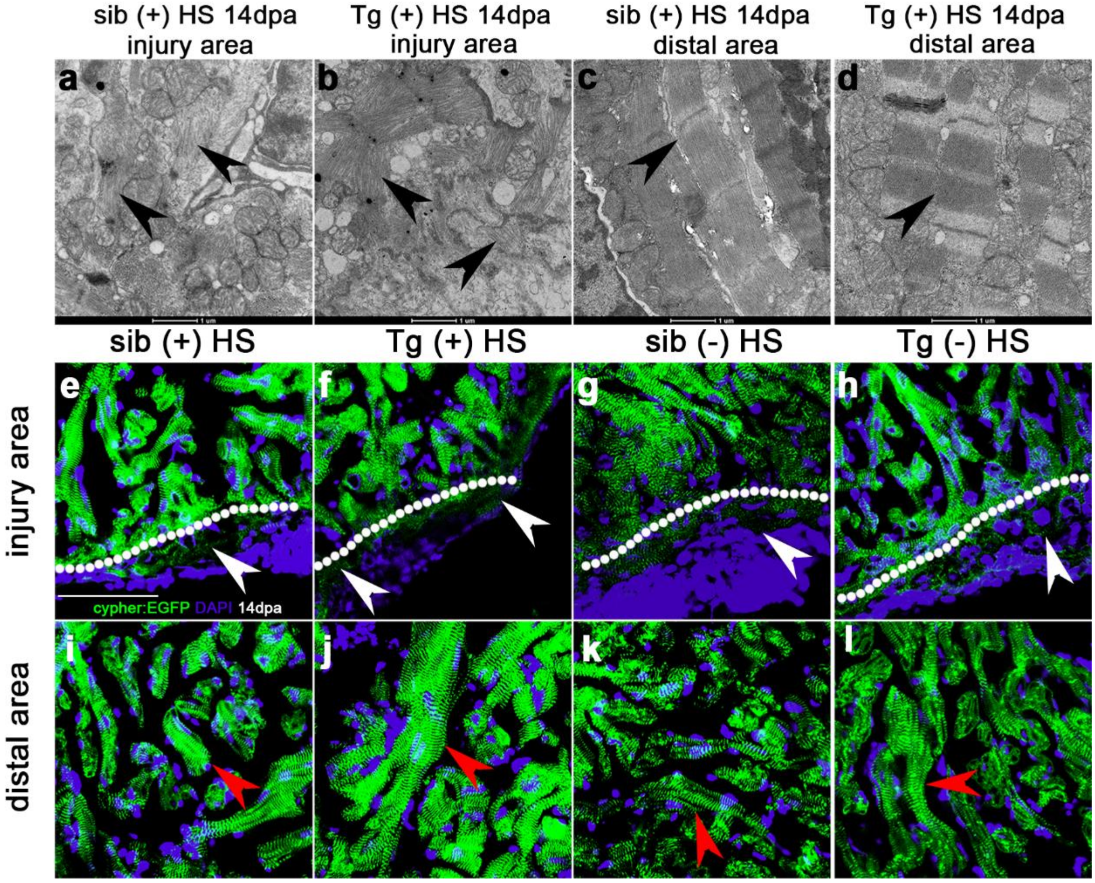

Supplementary Figure 7. Inhibition of Brg1 has no effect on cardiac sarcomere disassembly during heart regeneration. (a-d) Transmission electron microscopy images of myocytes of wt sibling (sib) (a, c), and Tg(*hsp70*:dn-xBrg1) transgenic (tg) heart (b, d) at 14 dpa. Note the normal sarcomere structures (black arrowhead) in distal cardiomyocytes from the injury site (c, d) and sarcomere disarray (black arrowhead) in cardiomyocytes in the injury site of both tg and wt hearts with heat shock treatment (a, b). Scale bars, 1μm. (e-h) Z-disks were labeled by using cypher-EGFP fusion protein. Cypher-EGFP-labeled sarcomere was all disarrayed in cardiomyocytes near the injury site (e-h), but was normal in distal area (i-l), of tg and wt sibling hearts at 14 dpa with or without heat-shock treatment. White arrowheads indicate sarcomere disarray (e-h) while red arrowheads indicate normal sarcomeres (i-l). DAPI co-stained for the nuclei; Scale bar, 50μm.

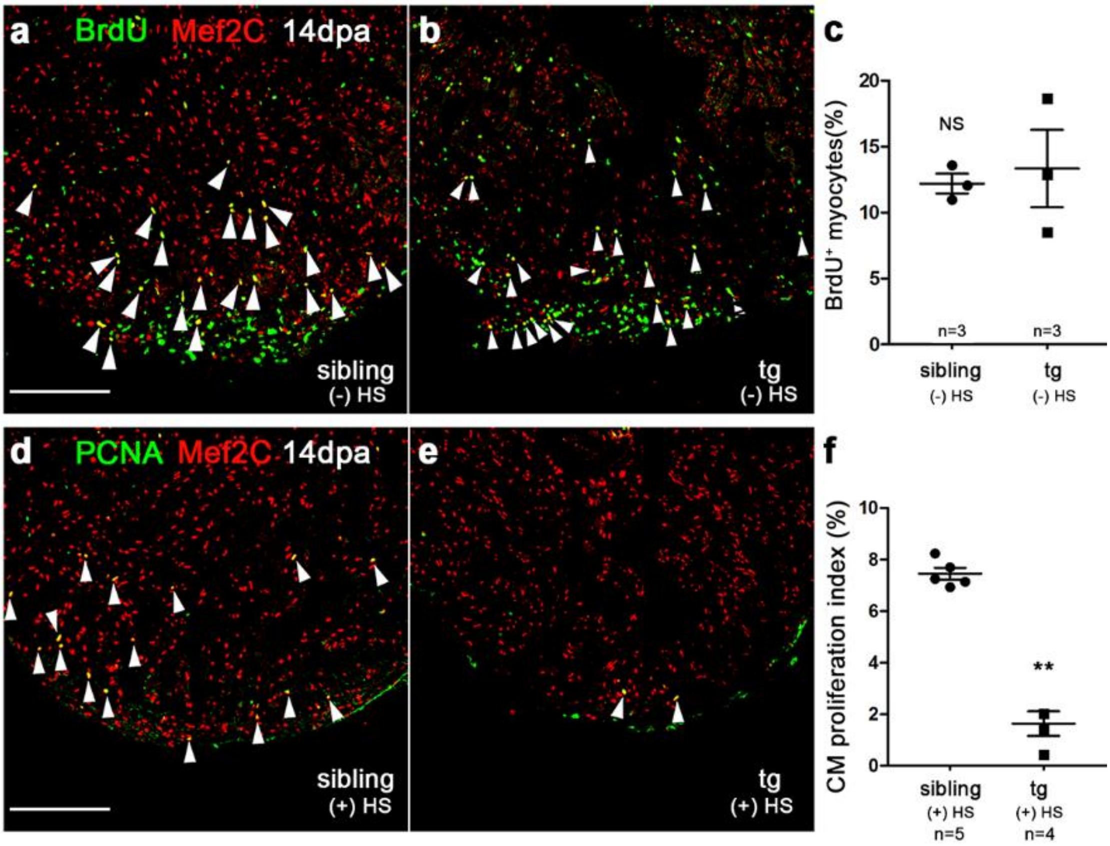

Supplementary Figure 8. Inhibition of *brg1* impairs cardiac regeneration. (a-c) $BrdU^+$/$Mef2C^+$ proliferating cardiomyocyte were comparable between wt sibling hearts (a) and dn-xBrg1 transgenic hearts (b) at 14 dpa without heat shock treatment [(-) HS]. (c) Percentages of $BrdU^+$/$Mef2C^+$ cardiomyocytes in the injured area. (d-f) $PCNA^+$/ $Mef2C^+$ proliferating cardiomyocytes (arrowheads) decreased in dn-xBrg1 tg hearts (e) compared with wt sibling hearts (d) at 14 dpa with heat shock treatment. (f) Quantification of cardiomyocyte proliferation assessed by $PCNA^+$/ $Mef2C^+$ staining Scale bars, 100 μm. Data presented are mean ± s.e.m.; paired Student's *t*-test, sample numbers are listed under each group, ** $p < 0.01$.

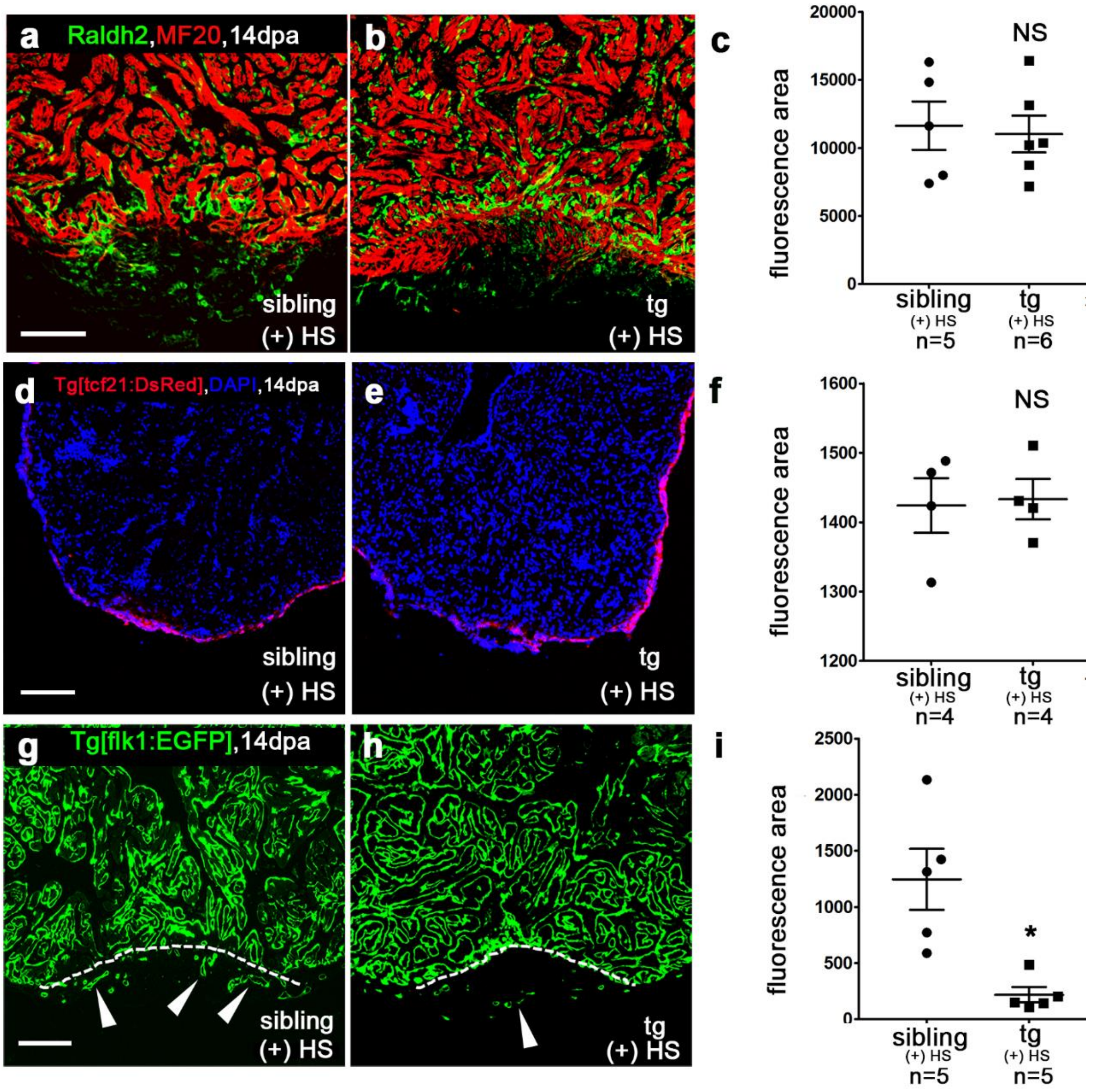

Supplementary Figure 9. (a-c) Levels of Raldh2 assessed by immunostaining with anti-Raldh2 (green) and anti-myosin heavy-chain (MF20) (red) were comparable in wild-type sibling (a) and Tg(*hsp70*:dn-xBrg1) (b) hearts at 14 dpa. (c) Quantification of the fluorescence intensity of Raldh2 signals in the original wound sites. (d-f) Tcf21:DsRed signal was comparable in Tg(*tcf21*:DsRed) control hearts (d) and Tg(*hsp70*:dn-xBrg1;*tcf21*:DsRed) transgenic hearts (e) at 14dpa. (f) Quantification of the fluorescence intensity of tcf21-DsRed signals was shown (g-i) Coronary vessels were reduced in Tg(*hsp70*:dn-xBrg1;*flk1*:EGFP) transgenic hearts (h) compared with those in wild-type Tg(*flk1*:EGFP) sibling hearts (g) at 14 dpa. The resection sites are marked with dashed lines. (i) Quantification of the fluorescence intensity of flk1-EGFP signals in the original wound sites was shown Heat shock was applied from 5 to 14 dpa. Scale bars, 100μm. Data presented are mean ± s.e.m., paired Student's *t*-test); NS, no significant difference; *p <0.05, sample numbers are listed under each group.

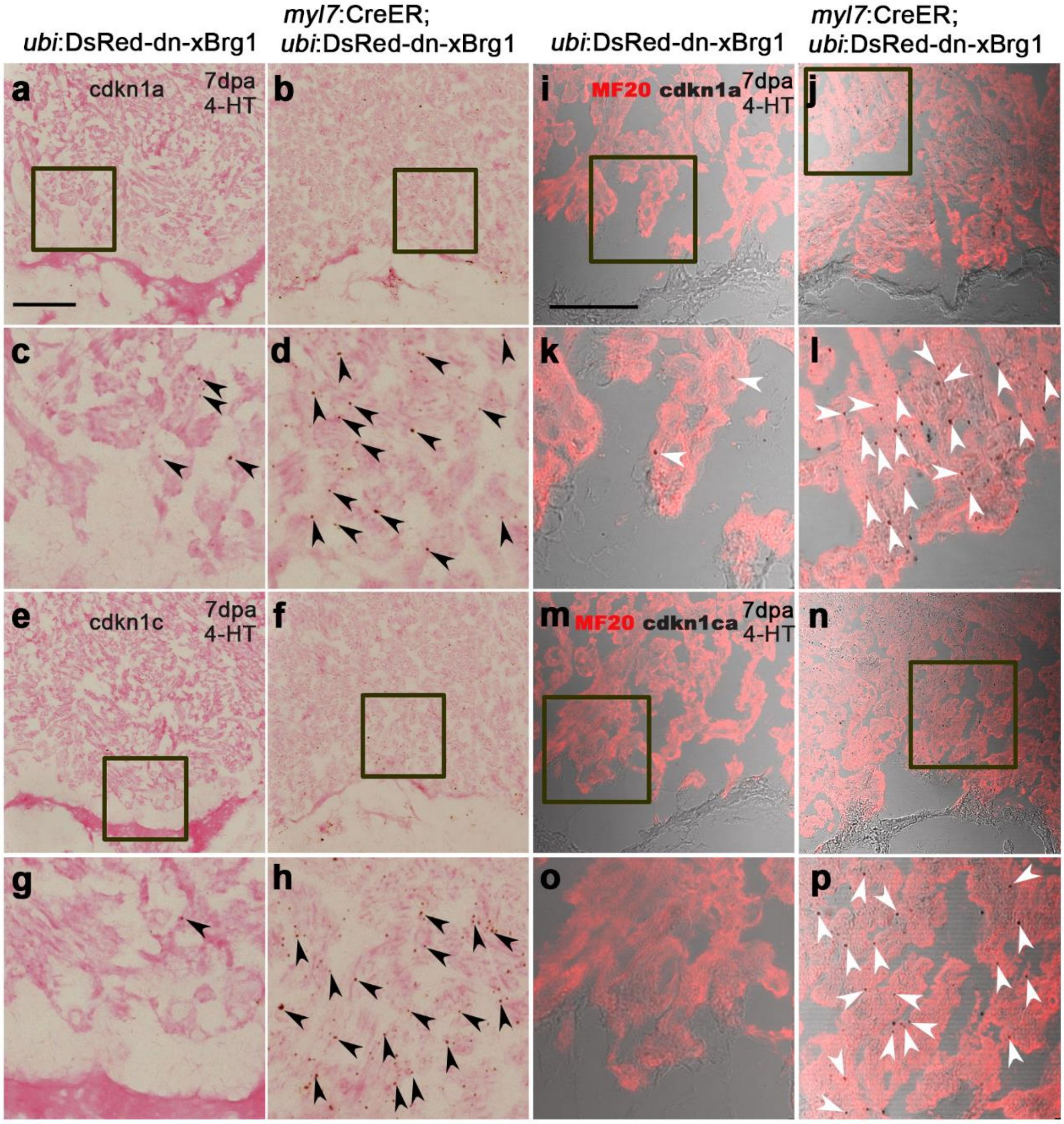

Supplementary Figure 10. *cdkn1a* and *cdkn1c* are induced in Tg(*myl7*:CreER; *ubi*:DsRed-dn-xBrg1) transgenic hearts. (a-h) RNAScope *in situ* hybridization analysis with *cdkn1a* (a-d) and *cdkn1c* (e-h) probes on frozen sections of injured control Tg(*ubi*:DsRed-dn-xBrg1) hearts (a, c, e, g) and injured Tg(*myl7*:CreER; *ubi*:DsRed-dn-xBrg1) transgenic hearts (b, d, f, h) at 7dpa after 4-HT induction. Panels c, d, g and h are high-magnification images of areas in squares in panels a, b, e and f. Black arrowheads indicate the RNAScope signals. (i-p) Bright-field views of *cdkn1a* (i-l) and *cdkn1ca* (m-p) expression by RNAscope *in situ* hybridization, which were merged with MF20 antibody confocal images, on frozen sections of injured Tg(*ubi*:DsRed-dn-xBrg1) hearts (i, k, m, o) and injured Tg(*myl7*:CreER; *ubi*:DsRed-dn-xBrg1) transgenic hearts (j, l, n, p) at 7dpa after 4-HT induction. Panels k, l, o and p are high-magnification images of areas in squares in panels i, j, m and n. White arrowheads show RNAScope signals in cardiomyocytes. Scale bars, 100 µm.

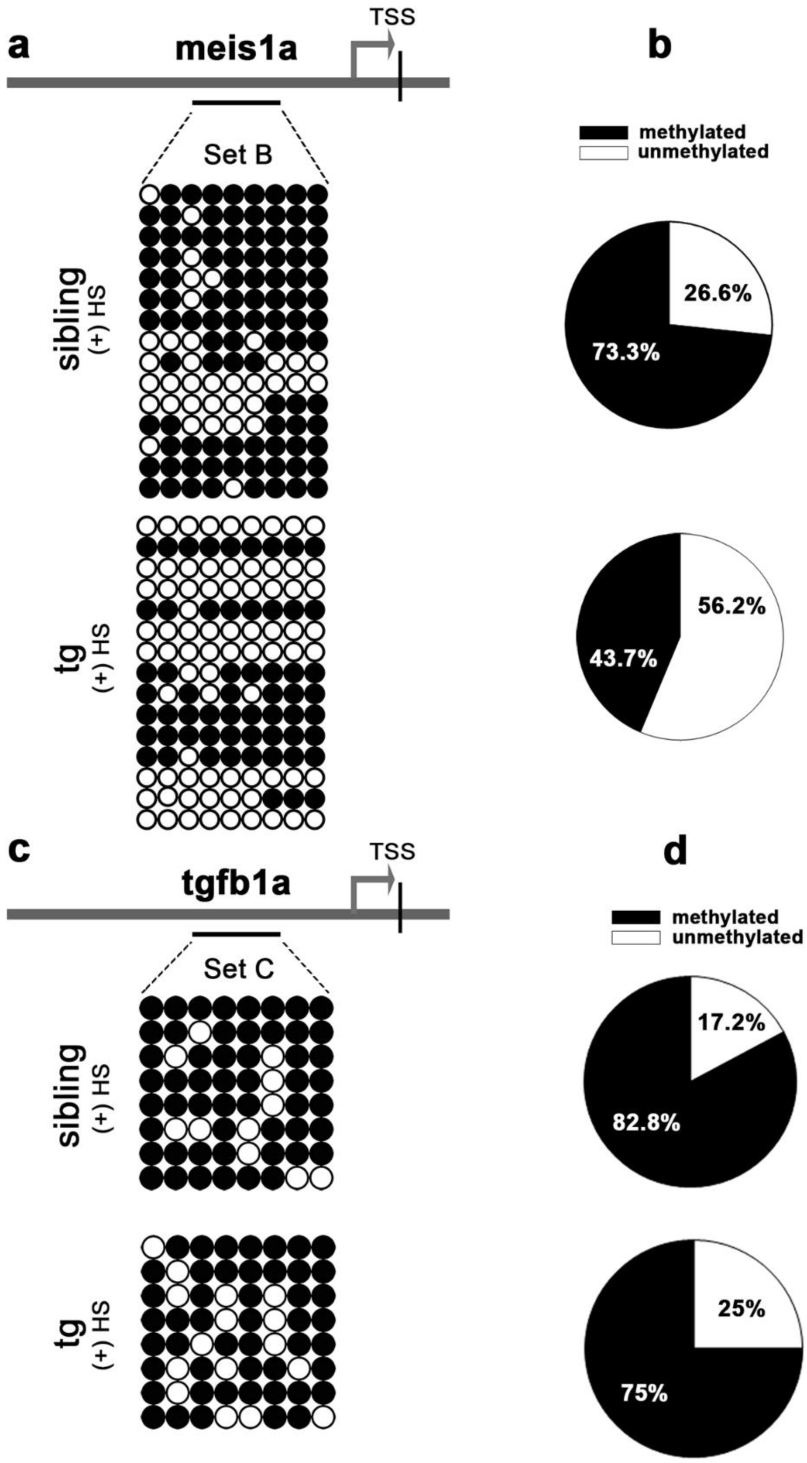

Supplementary Figure 11. (a) *meis1a* promoter methylation of 9 individual CpG sites (set B) of Tg(*hsp70*:dn-xBrg1) and wild-type sibling hearts after daily heat shock from 5 to 14 dpa. TSS, transcription start site; open circles, unmethylated CpG; filled circles, methylated CpG. (c) *tgfb1a* promoter methylation of 8 individual CpG sites (set C) of wild-type sibling and dn-xBrg1 transgenic (tg) hearts after daily heat shock from 5 to 14 dpa. The percentages of unmethylated (white) and methylated (black) DNA from panels a and c are shown in panels b and d. Note that both *meis1a* and *tgfb1a* promoters are less methylated in dn-xBrg1 transgenic hearts.

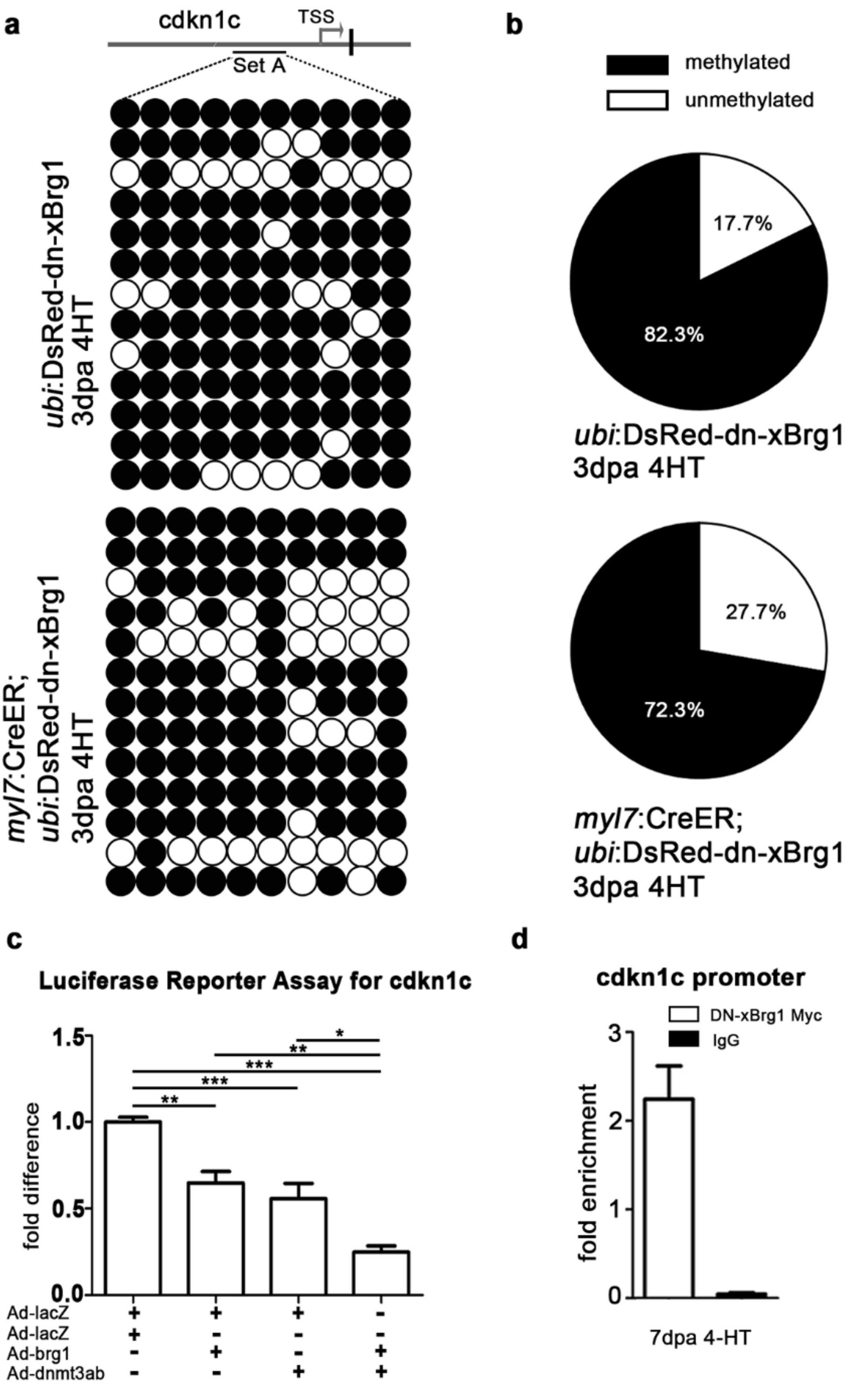

Supplementary Figure 12. (a, b) Methylation patterns of 10 individual CpG sites in the *cdkn1c* promoter of Tg(*myl7*:CreER; *ubi*:DsRed-dn-xBrg1) and control

Tg(*ubi*:DsRed-dn-xBrg1) transgenic hearts at 3 dpa. Upper panel, 10 CpG island sites (set A) of the *cdkn1c* promoter region and transcription start site (TSS); lower panels, *cdkn1c* methylation patterns of Tg(*ubi*:DsRed-dn-xBrg1) hearts and Tg(*myl7*:CreER; *ubi*:DsRed-dn-xBrg1) hearts at 3 dpa after 4HT induction, with open circles for "unmethylated" and filled circles for "methylated" CpG islands. Methylated DNA sequences were obtained by bisulfite sequencing. The percentages of unmethylated (white) and methylated (black) DNA from panels a are shown in panel b. (c) Luciferase reporter assays indicate that over-expression of zebrafish *brg1* and *dnmt3ab* suppressed the transcription of *cdkn1c* in cultured P4-rat neonatal cardiomyocytes. The P4-neonatal cardiomyocytes were transfected/infected with the indicated adenoviral constructs and luciferase reporter constructs (pREP4-cdkn1c-Luc and pREP4-renilla), and cells were collected and measured for luciferase activity at 24 h after transfection/infection. Equal amounts of adenovirus were used for each group. Firefly luciferase activity was normalized by *Renilla* luciferase activity (* $p<0.05$, ** $p<0.01$, *** $p<0.005$; data are mean ± s.e.m.; one-way ANOVA followed by Bonferroni's Multiple Comparison Test). (d) ChIP assays with anti-Myc antibody showed that DN-xBrg1-Myc bound to the *cdkn1c* promoter in Tg(*myl7*:CreER; *ubi*:DsRed-dn-xBrg1-Myc) hearts at 7dpa. Data are presented as Brg1 enrichment relative to control IgG.

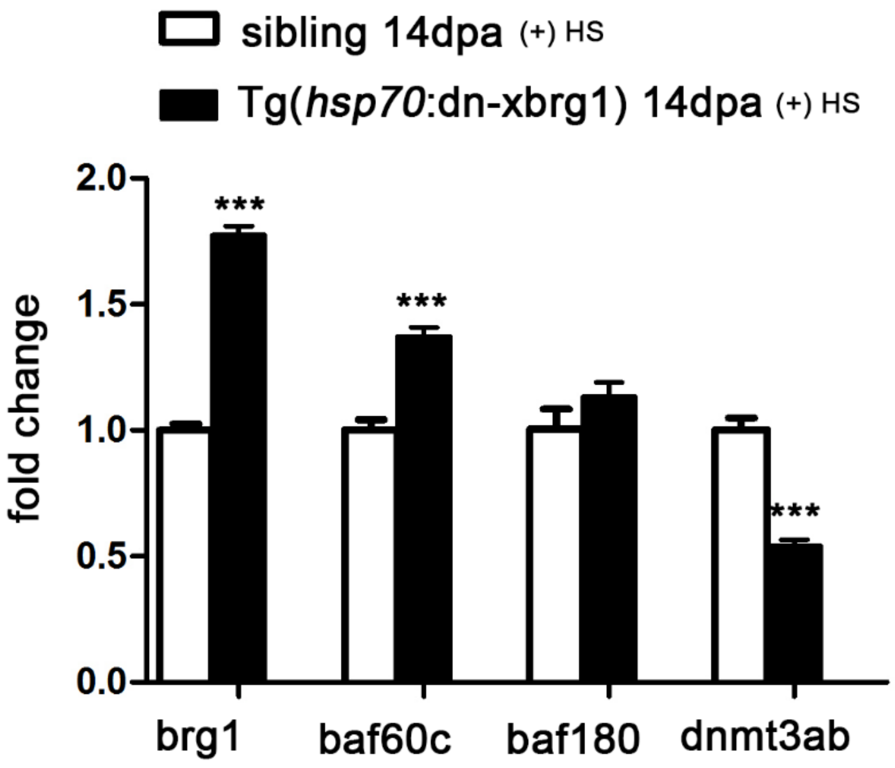

Supplementary Figure 13. Inhibition of Brg1 induces *brg1* and *baf60c* but represses *dnmt3ab*. RT-PCR revealed that *brg1* and *baf60c* were induced while *baf180* was not affected but *dnmt3ab* decreased in Tg(*hsp70*:dn-xbrg1) transgenic hearts compared with wild-type sibling hearts at 14dpa with heat shock from 5 to 14 dpa (***P <0.001; data presented are mean ± s.e.m.; paired Student's *t*-test).

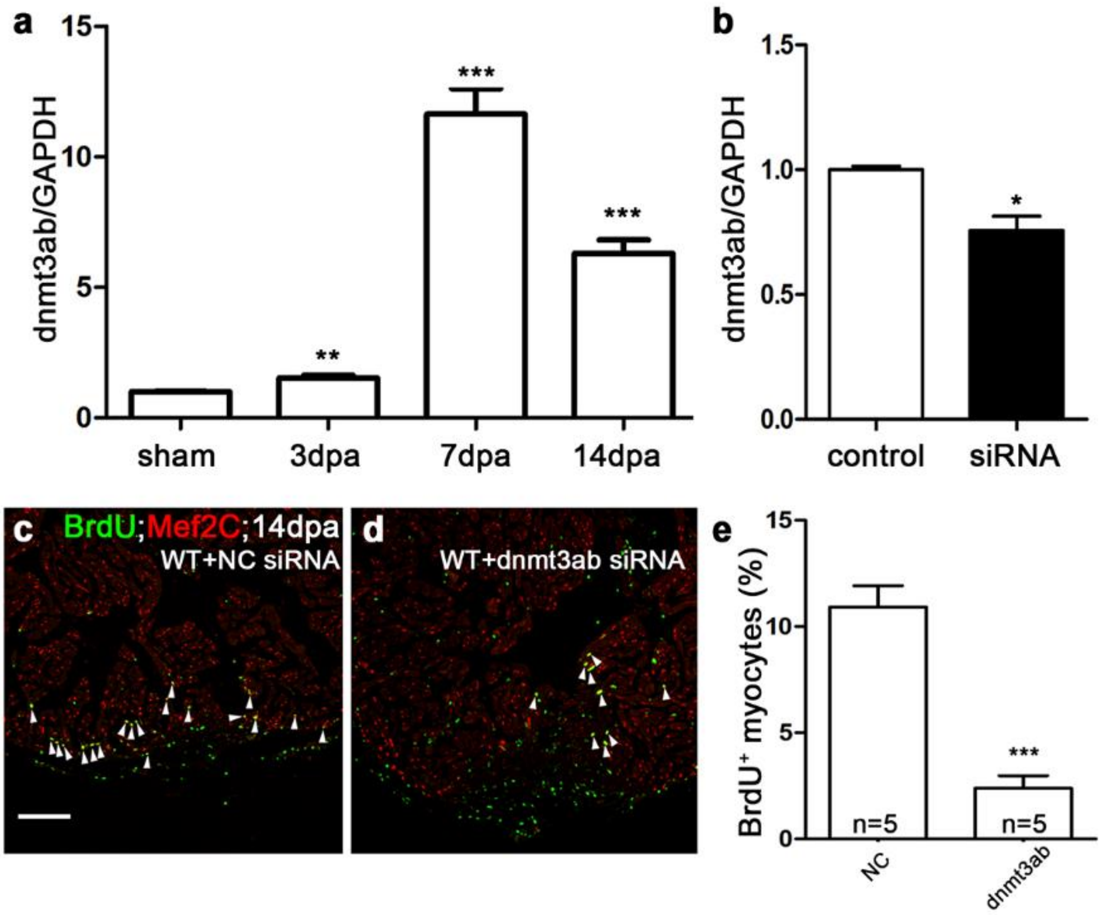

Supplementary Figure 14. Dnmt3ab is required for myocardial proliferation. (a) Quantitative PCR showed that *dnmt3ab* was induced in injured hearts at 3, 7, and 14 dpa compared with sham hearts (**P <0.01; ***P <0.001; data presented are mean ± s.e.m.; paired Student's *t*-test). (b) Quantitative PCR showed that nanoparticle-mediated *dnmt3ab* siRNA decreased the RNA level of *dnmt3ab* in wild-type hearts (*P <0.05; data presented are mean ± s.e.m.; unpaired Student's *t*-test). (c-e) BrdU $^{+}$/Mef2C^{+} proliferating cardiomyocytes decreased in dnmt3ab siRNA hearts (d) compared with negative control (NC) siRNA heart (c) at 14 dpa. The number (n) of hearts analyzed in each group is indicated in each bar (***P <0.001; data presented are mean ± s.e.m.; paired Student's *t*-test). Scale bar, 100µm.

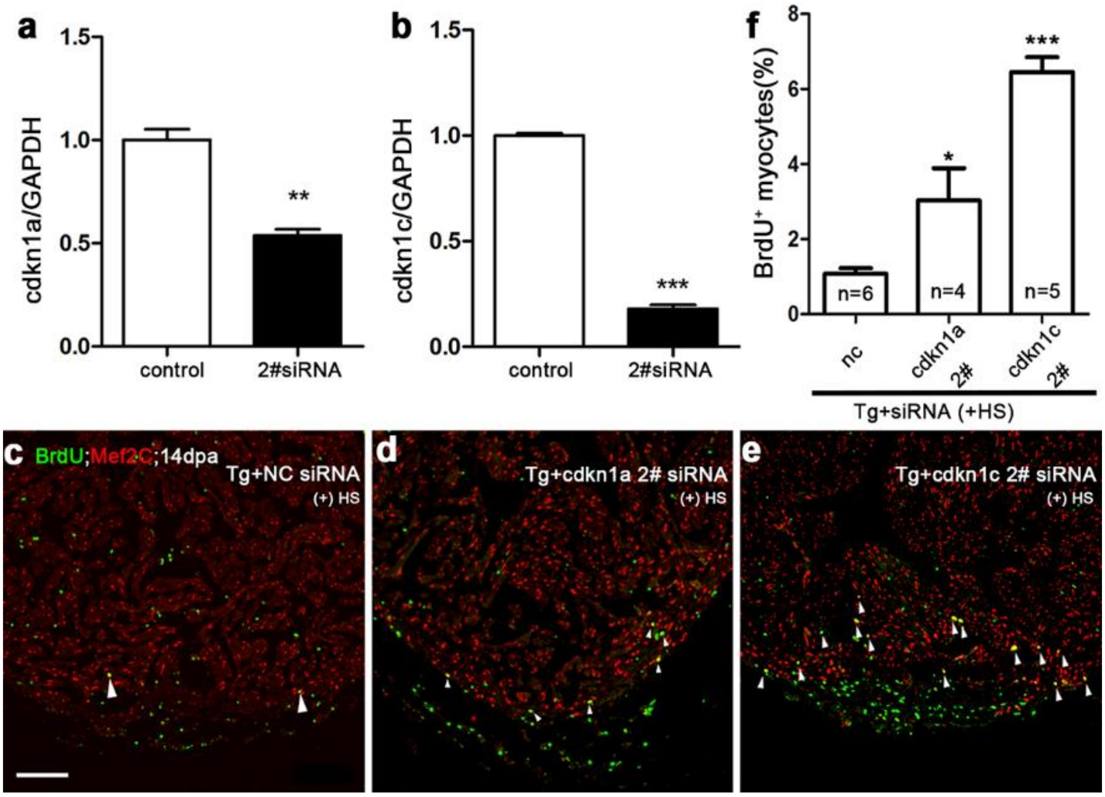

Supplementary Figure 15. Simultaneous siRNA knockdown of either *cdkn1a* or *cdkn1c* increases myocardial proliferation in dn-xBrg1 transgenic hearts. (a, b) Quantitative PCR showed that another independent siRNA for *cdkn1a* (a) or *cdkn1c* (b) decreased the RNA levels of *cdkn1a* and *cdkn1c* in wild-type hearts at 2 dpa. Control, *cdkn1a 2#* (a), or *cdkn1c 2#* (b) siRNA were injected at 1 dpa. The RNA level was normalized to GAPDH (**p <0.01, ***p < 0.001; data presented are mean ±s.e.m.; paired Student's *t*-test). (c-f) $BrdU^+/Mef2C^+$ proliferating cardiomyocytes increased in dn-xBrg1 transgenic hearts at 14 dpa injected with either *cdkn1a 2#* (d) or *cdkn1c 2#* (e) compared with control NC siRNA (c). Statistics of panels c-e is shown (f) (*p <0.05, ***p <0.001; data are mean ± s.e.m.; one-way ANOVA followed by Dunnett's Multiple Comparison Test; nc served as control). The number (n) of hearts analyzed in each group is indicated in each bar; heat shock was applied from 5 to 14 dpa. Scale bar, 100μm.

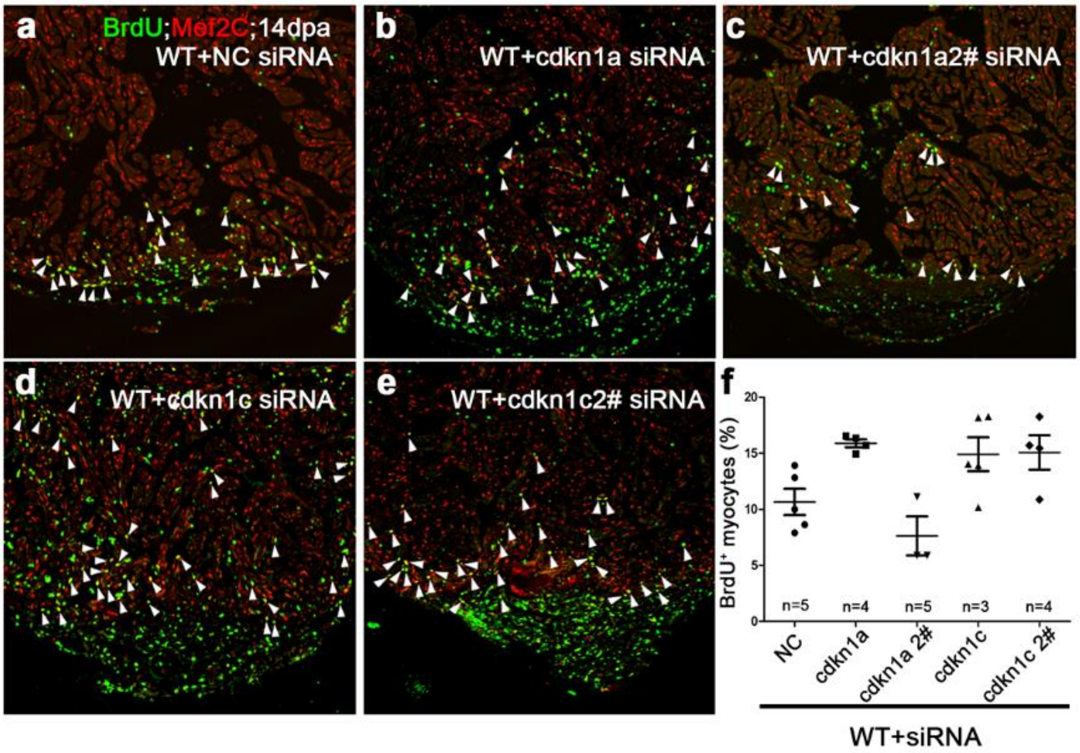

Supplementary Figure 16. Knockdown of CDK inhibitors in wild-type hearts had minimal effects on myocardial proliferation. $BrdU^+/Mef2C^+$ proliferating cardiomyocytes (white arrowheads) were comparable among negative control (NC) siRNA (a), *cdkn1a* siRNA (b), *cdkn1a* 2# siRNA (c), *cdkn1c* siRNA (d), or *cdkn1c* 2# siRNA-treated (e) wild-type hearts at 14 dpa, consistent with low-level of *cdkn1a* and *cdkn1c* in wild-type injured hearts. (f) Statistics of panels a-e. The number (n) of hearts analyzed in each group is indicated in each group. Scale bar, 100μm.

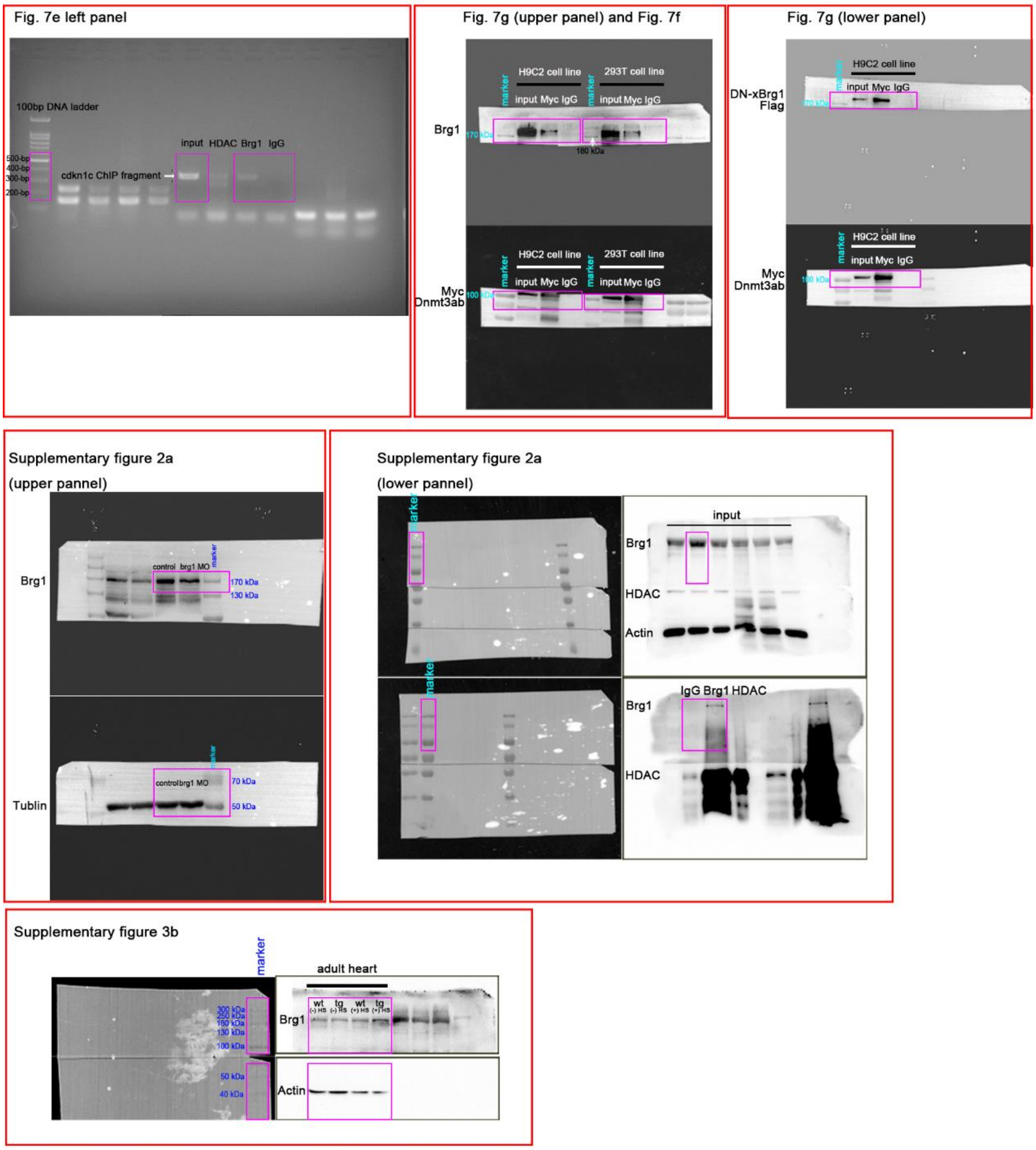

Supplementary Figure 17. Original un-cripped images of all the blots and gels that are shown in the Figures and Supplementary Figures. The pink boxes indicate the area/bands selected for the Figures or Supplementary Figures.

Supplementary Table 1. PCR primer sequences

brg1-F	ATGTCCACTCCTGACCCACCCATGGGCGGGAC
brg1-R	ATCTTCCTCGCTGCCACTAGCC
dnmt3ab-F	ATGAACTCAATGGAGGACCATGGCG
dnmt3ab-R	TTAAGTTCCGACGCAGGCGAAGTAC
cdkn1a-RT-F	GCTGCACTCCCGCATGAAGT
cdkn1a-RT-R	CACTAGACGCTTCTTGGCTTGGT
cdkn1ba-RT-F	TCAGCACGCCGAGGAAACGA
cdkn1ba-RT-R	CTGGCGAAGTAGTCGATGGTGAG
cdkn1bb-RT-F	ACGGGAATCACGACTGTAGGGTAA
cdkn1bb-RT-R	TCTGGGCGTTCGGGTCACTT
cdkn1c-RT-F	AGGCGATTTCAGAGGACACTTTGC
cdkn1c-RT-R	GGAAGCGTCTCCTGTTGCGTTAA
cdkn1d-RT-f	AGCTCTGCTGCATTTCGCATCTAT
cdkn1d-RT-r	AATGTCCTCCTCCTGCCTCTTCAA
Meis1a-RT-f	TTGGCAACAAATCTTCGCTTGGAA
Meis1a-RT-r	TCCTGGTCAGCTTTCGCAACAA
meis1b-RT-f	CCAATGTTCAATCCAGGAGATCCA
meis1b-RT-r	GCAGCATCCTCGTCTGTCCAT
meis2a-RT-f	CTTCATGTCTGATGAGCTAGTCCT
meis2a-RT-r	ACGCTGCGTTAATGATCGGAG
meis2b-RT-f	AGCACACATCTGACACAAATTCCA
meis2b-RT-r	ACTTAGCCTTACAAGAGCACTGTT
meis3-RT-f	TTCACGCTTCTGCTGCTACATTCT
meis3-RT-r	ACTGCACCAACTCCTCATACCTCT
baf60c-RT-f	GCTTGTTCCAGAGTCACAGGCATA
baf60c-RT-r	GCATCAGGCTTGGCAGCGTTA
baf180-RT-f	GGATGCTTCTGGATGCTGTGTTGA
baf180-RT-r	GTATTCGGTGTGCGATGCTCTTCA
col1a1a-RT-f	CCAGACGGCACCAAGAAGAACC
col1a1a-RT-r	GTTGACGCAAGTCTCGCCAGTT
col1a2-RT-f	GTGAAGATGGCAACAATGGCAGAC
col1a2-RT-r	AGGAAGACCACGACCACCTCTC
TGFb2-RT-f	CCACAGCGGTCAGTCTCCACAT
TGFb2-RT-r	GACAGGCTCCTGCACAGAAGTTG
TGFb3-RT-f	GCCGCTCACCATCCTCTACTAC
TGFb3-RT-r	GGACCGAACATTACACGCTACAG
vimentin-RT-f	AACCTGACCTGACCGCTGCT
vimentin-RT-r	CTGACGCTCCAGAGACTCATTCG
dnmt3ab-RT-f	ATGAACTCAATGGAGGACCATGGCG
dnmt3ab-RT-r	TTAAGTTCCGACGCAGGCGAAGTAC
cdkn1c-chip-F	GCAGCAGCTCCATGTCGATTCT
cdkn1c-chip-R	AGTTGGTCTTATGGTGGTGTAGGC
cdkn1c-promoter-f	GTTGGTCTTATGGTGGTGTAGGC
cdkn1c-promoter-r	AAGTTCAATAACAATATACCAA
brg1 probe F	GCCAGAGGAAGGAGGTGGATTAC
brg1 probe R	GGTCTTCCTCACTGTCGTCATCACT
baf60c probe F	GCAGCAGGCCGTACAGAACCGAAAC
baf60c probe R	AGGGTCGGGGGGCAGTAAAAGGTTG
baf180 probe F	CAATTAAGAAAGTGTTTGCCCAGAG
baf180 probe R	TGGGGTTTTTGATCTGATGGTAGT
cdkn1c-methy-f	TGTGTGTAAGACTCTACTTTATGTAACAAG
cdkn1c-methy-r	GAGGAACATACCCTCTGGATATCTC
tgfb1a-methy-f	TTTTTGATTTTTAAAGGTGTTTTAG
tgfb1a-methy-r	AACACAACACTTACTAACTAACCTCC
meis1a-methy-f	TGGTGTGTGTATTTGTGTGTTTTTA
meis1a-methy-r	CATAATCTCTACTCCCAAACTCCAA

Supplementary Table 2. siRNA sequences

	Sense（5'-3'）	Antisense（5'-3'）
cdkn1a siRNA	UCGACUUUGCGUCUGAGAATT	UUCUCAGACGCAAAGUCGATT
cdkn1a #2siRNA	CCUACGUUCACUCGGUAAUTT	AUUACCGAGUGAACGUAGGTT
cdkn1c siRNA	GCGACGUCUGUUAACGCAATT	UUGCGUUAACAGACGUCGCTT
cdkn1c #2siRNA	GCAGUGUUACAAUGUCUAATT	UUAGACAUUGUAACACUGCTT
dnmt3ab siRNA	GCCAACCUACAAUAAGCAATT	UUGCUUAUUGUAGGUUGGCTT
